# A machine learning model identifies patients in need of autoimmune disease testing using electronic health records

**DOI:** 10.1038/s41467-023-37996-7

**Published:** 2023-04-25

**Authors:** Iain S. Forrest, Ben O. Petrazzini, Áine Duffy, Joshua K. Park, Anya J. O’Neal, Daniel M. Jordan, Ghislain Rocheleau, Girish N. Nadkarni, Judy H. Cho, Ashira D. Blazer, Ron Do

**Affiliations:** 1 The Charles Bronfman Institute for Personalized Medicine, Icahn School of Medicine at Mount Sinai, New York, NY USA; 2 Medical Scientist Training Program, Icahn School of Medicine at Mount Sinai, New York, NY USA; 3 The BioMe Phenomics Center, Icahn School of Medicine at Mount Sinai, New York, NY USA; 4 Department of Genetics and Genomic Sciences, Icahn School of Medicine at Mount Sinai, New York, NY USA; 5 Department of Microbiology and Immunology, University of Maryland School of Medicine, Baltimore, MD USA; 6 Department of Medicine, Icahn School of Medicine at Mount Sinai, New York, NY USA; 7 Division of Rheumatology, Hospital for Special Surgery, New York, NY USA

**Keywords:** Rheumatic diseases, Machine learning

## Abstract

Systemic autoimmune rheumatic diseases (SARDs) can lead to irreversible damage if left untreated, yet these patients often endure long diagnostic journeys before being diagnosed and treated. Machine learning may help overcome the challenges of diagnosing SARDs and inform clinical decision-making. Here, we developed and tested a machine learning model to identify patients who should receive rheumatological evaluation for SARDs using longitudinal electronic health records of 161,584 individuals from two institutions. The model demonstrated high performance for predicting cases of autoantibody-tested individuals in a validation set, an external test set, and an independent cohort with a broader case definition. This approach identified more individuals for autoantibody testing compared with current clinical standards and a greater proportion of autoantibody carriers among those tested. Diagnoses of SARDs and other autoimmune conditions increased with higher model probabilities. The model detected a need for autoantibody testing and rheumatology encounters up to five years before the test date and assessment date, respectively. Altogether, these findings illustrate that the clinical manifestations of a diverse array of autoimmune conditions are detectable in electronic health records using machine learning, which may help systematize and accelerate autoimmune testing.

## Abstract (web summary)

Early diagnosis can significantly improve treatment options and prevent severe organ damage in individuals with autoimmune diseases. Here, the authors develop a machine learning model that uses electronic health records to identify patients with clinical suspicion of autoimmune diseases.

## Introduction

SARDs are a heterogeneous group of conditions with autoimmune dysregulation, such as systemic lupus erythematosus, rheumatoid arthritis, and inflammatory myositis, that cause characteristic systemic and musculoskeletal manifestations^1,2^. The prevalence of SARDs has been growing over the last several decades and is a significant burden on patients, their families, and healthcare systems^3–7^. Pathogenesis of SARDs is driven by a complex combination of environmental factors and genetic predisposition, resulting in heterogeneous clinical presentations and overlapping syndromes^8–10^. A lack of healthcare providers specializing in SARDs contributes to missed or delayed diagnoses in patients^11,12^. Consequently, undiagnosed patients endure long and difficult diagnostic journeys, sometimes taking years if not decades to receive a diagnosis^12–14^. During this time, many are misdiagnosed with symptoms commonly attributed to anxiety, mental illness, stress, or lifestyle factors^12^. Misdiagnosis and delayed diagnosis of SARDs lead to poorer clinical outcomes and greater mortality^14,15^.

In some individuals with SARDs, autoantibodies generated by plasma cells are key to pathogenesis and detectable for diagnostic purposes. These autoantibodies can target self-antigens, mark cells for immune-mediated destruction, impede cellular function, and incite inflammatory responses that cause tissue injury^16^. In individuals with suspected SARDs, serum autoantibody testing can help support a diagnosis^17^. For example, anti-citrullinated peptides (anti-CCP) and rheumatoid factor (RF) antibodies are useful in predicting and diagnosing rheumatoid arthritis^18,19^. When performed by primary care providers, autoantibody testing can serve as an important first step in the diagnostic workup that triggers a referral to rheumatology for a thorough SARD evaluation^20,21^. These steps are critical for a timely diagnosis of SARDs and to initiate morbidity- and mortality-reducing treatment; thus, support for timely diagnosis remains a major unmet need^22,23^. A systematic data-driven approach^24^ to identify individuals with high suspicion for SARDs who would benefit from autoantibody testing and rheumatology consultation could improve the diagnosis and care of patients.

Vital signs, laboratory test results, medications, symptoms, and other clinical features in EHRs represent a patient’s health profile that may reveal a need for diagnostic testing of SARDs. Health systems accrue millions of these clinical data points in the EHR over time; data-driven approaches with artificial intelligence enable the analysis and interpretation of this vast dataset^25–31^. Machine learning models trained on EHR data have recently been shown to accurately predict risk of coronary artery disease^32,33^. A similar approach has been used to prioritize patients for genetic testing^26^. Given that many SARDs phenotypes are expressed in a multi-systemic manner with variability over time, longitudinal EHR data could be invaluable for training a model to prioritize individuals for testing^34^. We reasoned that this distinct pattern of immune-driven disease manifestations is indicative of SARDs and can be detected by analyzing EHRs with machine learning.

Here, we asked whether a machine learning model using longitudinal and multimodal EHR data can identify patients with a clinical profile characteristic of receiving rheumatological evaluation for suspected SARDs. The output of existing machine learning models typically arrives at the endpoint of the diagnostic workflow (disease diagnosis)^24^, predicting case-control labels with inherent biases and inaccuracies while replacing human decision-making^27,35–37^. Instead, we applied a model to decision-making points embedded in the workflow itself^25,26,38^ (autoantibody testing and rheumatologist involvement) that captures clinical suspicion of SARDs. We trained and tested our model using EHRs from 161,584 individuals across two institutions with autoantibody and rheumatology data, and targeted a core group of SARDs comprising ANCA-associated vasculitis, antiphospholipid syndrome, dermatomyositis, diffuse and limited cutaneous systemic sclerosis, drug-induced lupus, mixed connective tissue disease, polymyositis, rheumatoid arthritis, Sjogren syndrome, and systemic lupus erythematosus. The model identified more individuals with autoantibodies and autoimmune disease diagnoses than current clinical standards, and accurately predicted the need for autoantibody and rheumatologist testing up to 5 years before the actual testing date. This proof-of-concept study and these findings provide evidence that individuals in need of rheumatic disease evaluation can be identified systematically by harnessing artificial intelligence trained on large-scale EHR data.

## Results

### Study population

**Fig. 1:**
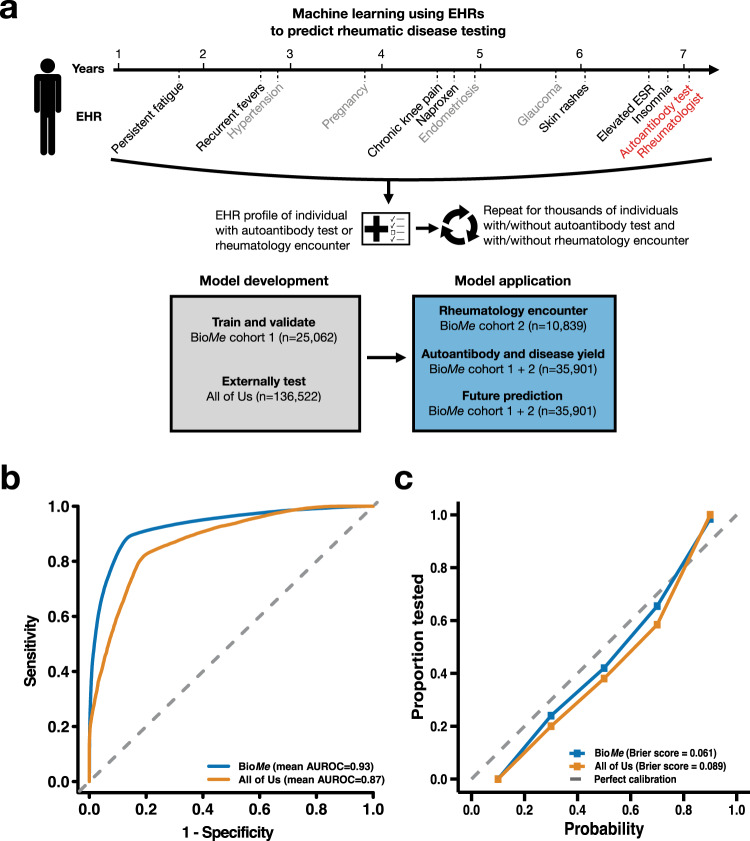
Model performance to predict autoantibody testing using electronic health records (EHRs) in validation and external test datasets. **a** Schematic of study design depicting a hypothetical individual who received autoantibody testing and had a rheumatology encounter; EHR data preceding the test or encounter date is collected as input to the machine learning model. **b**, **c** Performance metrics in the validation dataset from Bio*Me* Biobank (Bio*Me* cohort 1) and the external test dataset from All of Us.

After filtering and quality control (Methods), the study population included 161,584 participants from three cohorts across two institutions (Table 1). The model was trained and validated using EHR data from 25,062 participants in the Bio*Me* Biobank (Bio*Me*) cohort 1 (median [IQR] age, 60 [24] years; 15,091 [60%] female; 17,958 [72%] non-European ethnicity), comprising 6171 (25%) individuals who had received autoantibody testing. An external dataset of 136,522 EHRs from participants in All of Us (median [IQR] age, 61 [24] years; 85,196 [62%] female; 62,199 [46%] non-European ethnicity) was used for external testing, including 19,264 (14%) individuals who had been tested for autoantibodies. An independent dataset of 10,839 EHRs from participants in Bio*Me* cohort 2 (median [IQR] age, 56 [27] years; 6243 [58%] female; 7383 [68%] non-European ethnicity) was used for clinical applications of the model. The study was conducted in two phases: to first train and test the model using 161,584 participants from Bio*Me* cohort 1 and All of Us, then secondly to apply the model using 35,901 participants from Bio*Me* cohorts 1 and 2 (Fig. 1a). In all phases, the model targeted autoantibody tests with high specificity for a particular SARD^2,17–19,39^ and not autoantibody tests with low specificity such as anti-nuclear antibody^40^.

**Table 1:** Summary of participant demographics and health system interactions

Trait	Bio*Me* Biobank cohort 1		All of Us		Bio*Me* Biobank cohort 2	
	Autoantibody tested (*n* = 6171)	Not tested (*n* = 18,891)	Autoantibody tested (*n* = 19,264)	Not tested (*n* = 117,258)	Rheumatology encounter (*n* = 1564)	No rheumatology encounter (*n* = 9275)
Age, median (IQR) years	62 (20)	60 (25)	62 (21)	61 (24)	59 (23)	55 (28)
Male, *n* (%)	1816 (29)	8155 (43)	5589 (29)	45,737 (39)	570 (30)	402 (45)
Ethnicity, *n* (%)						
African	1731 (28)	4805 (25)	4003 (21)	12,703 (20)	368 (20)	1512 (17)
European	1438 (23)	5666 (30)	10,747 (56)	34,276 (54)	458 (24)	2998 (33)
Hispanic	2544 (41)	6615 (35)	3212 (17)	11,633 (18)	784 (42)	2819 (31)
Other	458 (7.4)	1805 (9.6)	1302 (6.8)	4628 (7.3)	260 (14)	1641 (18)
Interactions with health system						
Unique ICD-10 codes, median (IQR)	66 (70)	31 (42)	109 (105)	56 (62)	61 (52)	29 (31)
Duration, median (IQR) years	11 (5.6)	9.2 (6.5)	14 (12)	9.1 (9.4)	7.1 (2.6)	5.6 (4.7)
Encounters, median (IQR)	106 (143)	41 (72)	168 (221)	75 (104)	76 (87)	31 (45)

Ethnicity, self-reported ethnicity; Other, self-reported ethnicity other than the listed ones, *ICD-10,* International Classification of Diseases 10, Duration, length of the electronic health record.

The presence of autoantibody testing in a patient’s medical history, regardless of it being positive or negative, indicates a strong clinical suspicion for SARDs diagnosis^16,17,21^. We examined the prevalence of SARDs diagnoses in the autoantibody-tested group versus the non-tested group as a validity check. In all three cohorts, the prevalence of SARDs diagnoses was greater in the tested group compared to the non-tested group: 1360 out of 6171 (22%) versus 245 out of 18,891 (1.3%) in Bio*Me* cohort 1 (*P* < 2.0 × 10^−308^), 3882 out of 19,264 (20%) versus 3402 out of 117,258 (2.9%) in All of Us (*P* < 2.0 × 10^−308^), and 391 out of 1870 (21%) versus 517 out of 8969 (5.1%) in Bio*Me* cohort 2 (*P* = 2.9 × 10^−95^), respectively.

### Training and validation of a model to predict autoantibody testing in BioMe cohort 1

We investigated whether a machine learning model could, in a systematic and high-throughput manner, differentiate between individuals who had received autoantibody testing and those who had not in order to capture clinical suspicion of SARDs. Features provided to the random forest-based model included the presence or absence of diagnosis codes and medications (binary data), and values of laboratory results and vitals (continuous data) (Methods). Diagnosis codes for SARDs and antibody testing (Z01.84) were removed to mitigate data leakage (Supplementary Table 1), as well as methotrexate, hydroxychloroquine, and azathioprine. We used a random sample of 90% of autoantibody-tested individuals and an equal number of non-tested controls for training (5213 tested and 5213 non-tested), and the remaining 10% of autoantibody-tested individuals and an equal number of non-tested individuals for validation (579 cases and 579 controls) iterated 100 times to reduce sampling bias; performance metrics were reported as the mean and 95% CI across all 100 iterations (Methods). In the validation dataset, the model predicted autoantibody testing with an area under the receiver operating curve (AUROC) of 0.93 (95% CI, 0.93–0.93), the accuracy of 0.89 (95% CI, 0.88–0.89), the sensitivity of 0.90 (95% CI, 0.90–0.90), and specificity of 0.87 (95% CI, 0.87–0.88) (Fig. 1b and Table 1). The prevalence of autoantibody testing was 23% in the validation dataset, with a negative predictive value (NPV) of 0.90 (95% CI, 0.89–0.90) and positive predictive value (PPV) of 0.88 (95% CI, 0.87–0.88). The model was calibrated with a Brier score of 0.061 upon monotonic regression (Fig. 1c). The most important features comprised symptoms, findings, and markers observed in the inflammatory response of SARDs, such as temperature, erythrocyte sedimentation rate, albumin level, white blood cell counts, and transferrin saturation^41^ (Supplementary Table 2). Analysis of the model’s interpretability with SHAP values revealed these features’ contributions to the model’s predictions in the direction expected with their biological effects (Supplementary Fig. 1).

### External testing of the model in All of Us

We sought to test the model in an external cohort from a different institution consisting of 136,522 participants (19,264 autoantibody-tested cases and 117,258 non-tested controls) selected with the same criteria used for the training and internal validation cohort (Table 1 and Methods). In this external testing dataset, the model predicted autoantibody testing with a similar classification performance as the internal validation: AUROC of 0.87 (95% CI, 0.87–0.88), accuracy of 0.82 (95% CI, 0.82–0.82), sensitivity of 0.82 (95% CI, 0.82–0.83), and specificity of 0.82 (95% CI, 0.81–0.82) (Table 2 and Fig. 1b). The prevalence of autoantibody testing was 14% and the model demonstrated a NPV of 0.82 (95% CI, 0.82–0.82) and PPV of 0.82 (95% CI, 0.82–0.82). The model was calibrated with a Brier score of 0.090 after monotonic regression (Fig. 1c).

**Table 2:** Performance of machine learning model to identify individuals who received autoantibody testing in the validation and external test datasets

Dataset	Total *n*	Autoantibody tested, *n* (%)	AUROC (95% CI)	Sensitivity (95% CI)	Specificity (95% CI)	Accuracy (95% CI)	NPV (95% CI)	PPV (95% CI)	F1 score
Validation (Bio*Me* cohort 1)	25,062	5792 (23)	0.93 (0.93 − 0.93)	0.90 (0.90 − 0.90)	0.87 (0.87 − 0.88)	0.89 (0.88 − 0.89)	0.90 (0.89 − 0.90)	0.88 (0.87 − 0.88)	0.89 (0.88 − 0.89)
External test (All of Us)	136,522	19,264 (14)	0.87 (0.87 − 0.88)	0.82 (0.82 − 0.83)	0.82 (0.81 − 0.82)	0.82 (0.82 − 0.82)	0.82 (0.82 − 0.82)	0.82 (0.82 − 0.82)	0.83 (0.82 − 0.82)

*n* number, *AUROC* area under the receiver operating characteristic curve, *NPV* negative predictive value, *PPV* positive predictive value.

### Sensitivity analyses of model

We conducted a series of sensitivity analyses to bolster the validity and clinical applicability of the model. First, the model was evaluated in a cohort design to guard against temporal bias whereby it was trained on EHRs with rolled-up diagnosis codes and medications of participants in a given year to predict autoantibody testing in the subsequent year and demonstrated similar performance as the primary model in both the internal validation and external test cohorts (Supplementary Fig. 2 and Supplementary Table 3). Second, the model was examined in a non-biobank cohort of 839,188 individuals from the Mount Sinai Data Warehouse (MSDW; median [IQR] age, 54 [33] years; 492,662 [59%] female; 410,613 [49%] non-European ethnicity) with 67,565 (8.1%) who had received autoantibody testing, and had a comparable performance with that in the other datasets (Supplementary Fig. 3 and Supplementary Table 4). Third, the model was assessed in a subgroup of individuals less than or equal to 50 years old and showed similar performance as in the whole population (Supplementary Fig. 4 and Supplementary Table 5).

### Validation in individuals with rheumatology encounter in Bio*Me* cohort 2

Autoantibody tests are an important diagnostic tool but do not account for all rheumatic disease assessment in a hospital system. We, therefore, aimed to validate the model in an independent set of individuals (Bio*Me* cohort 2) with cases defined as having an encounter with a rheumatologist (e.g., rheumatology consultation or treatment). Out of 10,839 participants selected with the same criteria as the training/internal validation cohort, there were 1564 cases with rheumatology encounters and 9275 controls with no evidence of being seen or treated by rheumatology (Supplementary Table 6 and Methods). SARDs diagnosis was observed in 416 out of 1564 (27%) individuals in the rheumatology encounter group versus in 512 out of 9275 (5.7%) in the non-encounter group (*P* = 3.9 × 10^−121^). The model demonstrated a similar classification performance in this rheumatology dataset compared to the autoantibody test datasets with an AUROC of 0.88 (95% CI, 0.88–0.88), accuracy of 0.82 (95% CI, 0.82–0.82), sensitivity of 0.82 (95% CI, 0.82–0.82), and specificity of 0.82 (95% CI, 0.81–0.82) (Supplementary Fig. 6). The prevalence of rheumatology encounters in the independent dataset was 14% and the model demonstrated a NPV of 0.82 (95% CI, 0.82–0.82), PPV of 0.82 (95% CI, 0.82–0.82). Calibration was measured with a Brier score of 0.098.

### Validation in individuals with autoantibodies and SARDs diagnoses

**Fig. 2:**
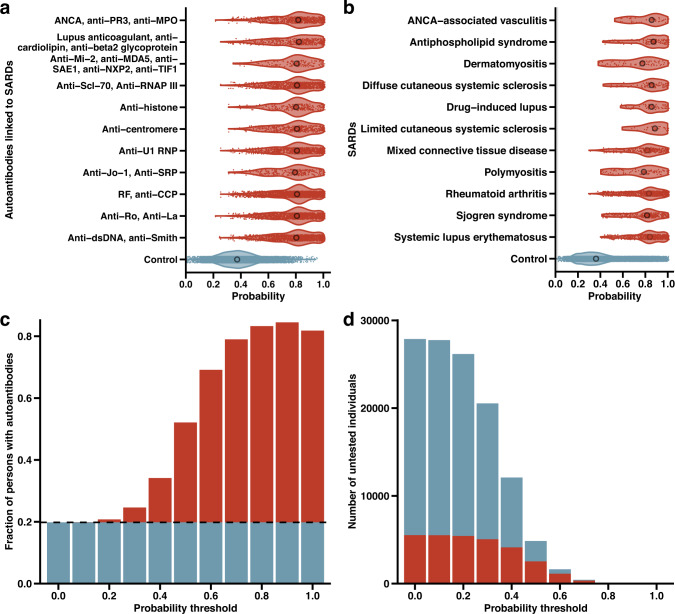
Autoantibodies and SARDs identified in the Bio*Me* Biobank. **a** Model-derived probabilities of autoantibody testing for 2748 participants who had autoantibodies corresponding to SARDs and a rheumatology encounter (red violin plots), and 20,487 controls who were not tested for autoantibodies and did not have a rheumatology encounter (blue violin plot). Median probabilities were compared in autoantibody-tested and untested individuals with Mann–Whitney’s *U*-test. **b** Probabilities of autoantibody testing for 2026 participants with SARDs diagnoses (red violin plots) and 32,979 controls without a SARDs diagnosis or autoantibody test (blue violin plots). Median probabilities were compared in cases and controls with Mann–Whitney’s *U-*test. **c** Fraction of individuals with autoantibodies identified by the model at increasing probability thresholds. The dashed line and blue portion of the bar plots represent the baseline fraction of autoantibodies detected in the population (0.20; 4754 out of 25,062), while the red portion of the bar plots indicate the excess fraction of autoantibodies identified by the model at each probability threshold. **d** Absolute number of individuals who have not been tested for autoantibodies at increasing probability thresholds; the red portion of the bar plots represents those expected to carry autoantibodies at each probability threshold. At thresholds of ≥0.8 and ≥0.9, 66 out of 77 and 13 out of 15 untested individuals are expected to have autoantibodies, respectively; there were 0 untested individuals at a threshold of 1.0.

Individuals with autoantibodies and individuals with SARDs diagnoses were identified among 35,901 participants from Bio*Me* cohorts 1 and 2 (Methods). We evaluated the model’s ability to detect 2748 individuals who had autoantibodies corresponding to SARDs and a rheumatology encounter (Fig. 2a). The median probability from the model output for this group with autoantibodies was 0.80 (IQR, 0.18) and was greater than the median probability of 0.38 (IQR, 0.17) in 20,487 controls without autoantibodies or rheumatology encounters (*P* < 2.0 × 10^−308^). Out of 8498 positive autoantibody tests, the most prevalent were RF and anti-CCP (rheumatoid arthritis; 2389 positive autoantibody tests [26% of all positive tests]), anti-Ro and anti-La (Sjogren syndrome; 1391 [15%]), and anti-dsDNA and anti-Smith (systemic lupus erythematosus; 1367 [15%]). Across all SARDs represented by the autoantibodies, individuals with autoantibodies had higher probabilities than controls. This ranged from a median probability of 0.78 (IQR, 0.19) for individuals with anti-Jo-1 and anti-SRP (polymyositis; *P* = 8.4 × 10^−151^) to 0.81 (IQR, 0.20) for individuals with lupus anti-coagulant, anti-cardiolipin, and anti-β2 glycoprotein (antiphospholipid syndrome; *P* = 9.8 × 10^−242^). These findings were replicated in All of Us, with probabilities in carriers of autoantibodies consistently higher than that in controls (Supplementary Fig. 7). In both Bio*Me* and All of Us, individuals with autoantibodies had higher probabilities than controls in subgroups stratified by sex, ethnicity, and education (Supplementary Fig. 5).

Since not all individuals with autoantibodies will have disease^17–21^, we further assessed the model’s performance to identify 2026 individuals with a clinical diagnosis of SARDs (Fig. 2b). The median probability from the model was greater for individuals with a SARDs diagnosis (0.81; IQR, 0.22) compared to 32,979 controls without a SARDs diagnosis or autoantibody test (0.35; IQR, 0.23) (*P* < 2.0 × 10^−308^). Out of 2691 SARDs diagnoses, the most prevalent were diagnoses of rheumatoid arthritis (923 [34%]), systemic lupus erythematosus (601 [22%]), and Sjogren syndrome (357 [13%]). Probabilities were higher across all SARDs compared to controls, ranging from a median probability of 0.77 (IQR, 0.25) for individuals diagnosed with dermatomyositis (*P* = 1.7 × 10^−29^) to 0.88 (IQR, 0.23) for individuals diagnosed with limited cutaneous systemic sclerosis (*P* = 1.3 × 10^−16^). In All of Us, these results were similar with greater probabilities for diagnosed individuals compared to controls (Supplementary Fig. 7). Individuals with SARD diagnoses had greater probabilities than controls in subgroups stratified by sex, ethnicity, and education in both Bio*Me* and All of Us (Supplementary Fig. 5).

We then sought to determine the fraction of individuals with potentially diagnosable SARDs detected by the model as compared to current clinical practice, and the number of undiagnosed individuals that could be identified. Potentially diagnosable SARDs was defined as individuals carrying autoantibodies specific for SARDs evidenced by a positive autoantibody test (Methods). This included 6684 out of 35,901 (19%) of participants with autoantibodies. The fraction and number of individuals with an autoantibody was calculated at different probability thresholds. The fraction of potentially diagnosable individuals grew with increasing probability thresholds to a maximum of 86% for higher probability thresholds (Fig. 2c). In addition, there were tens of thousands of potentially undiagnosed individuals (had not been tested for autoantibodies despite high EHR suspicion for SARDs) in the population who the model suggested may benefit from autoantibody testing (Fig. 2d). At a probability threshold of ≥0.5, 4838 individuals had not been tested for autoantibodies, of whom 51% (2463 individuals) would be expected to carry an autoantibody based on the model that could lead to a rheumatology evaluation and/or diagnosis. At an even higher threshold of ≥0.9, 15 individuals had not been tested, of whom 87% (13 individuals) would be expected to harbor autoantibodies. These trends were similarly observed in All of Us: 46% of non-tested individuals (12,262 individuals) would be expected to carry autoantibodies at a threshold of ≥0.5 and 95% of non-tested individuals (18 individuals) would be expected to carry autoantibodies at a threshold of ≥0.8 (Supplementary Fig. 7).

### Diverse autoimmune conditions captured by the model

**Fig. 3:**
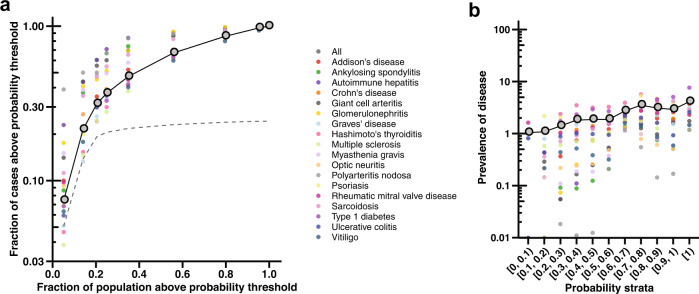
Model performance in capturing diverse autoimmune conditions. **a** Fraction of cases diagnosed with one of 18 autoimmune conditions above a probability threshold on a base-10 logarithmic scale versus the fraction of individuals that are tested for autoantibodies at the equivalent probability threshold. The dashed line marks the fraction of tested individuals above each probability threshold. The gray point indicates the mean across all autoimmune conditions. The columns of points represent values at different probability thresholds decreasing from left (≥0.9) to right (≥0.1) by increments of 0.1; the most lenient threshold (≥0.1) at the farthest right column of points yields the greatest fraction of individuals with autoimmune conditions detected by the model and the greatest fraction of the population tested. **b** Prevalence of autoimmune conditions on a base-10 logarithmic scale in different strata of probabilities increasing from 0 to 0.1 [0, 0.1) through 1.

Several autoimmune conditions were not included in the training of our model because they are not SARDs or an autoantibody test would not be an appropriate or informative modality for their diagnosis. To evaluate our hypothesis more extensively, we assessed the model’s capacity to identify individuals diagnosed with a wide array of 18 autoimmune conditions not part of the original training set (Methods). These were selected on the basis of the representation of different body systems and prevalence in the population, and clinical presentation that is readily analyzable in the EHR. A total of 6200 individuals across Bio*Me* cohorts 1 and 2 had a clinical diagnosis of at least one autoimmune condition. The model predicted two- to five-fold more cases of autoimmune conditions compared to that expected with the population rate of testing at different probability thresholds (Fig. 3a). For instance, 2774 (45%) cases of autoimmune conditions had a probability ≥0.5, while just 20% of the population would be tested for autoantibodies at this threshold, yielding a 2.3-fold increase in identified cases. The model increased the yield of cases the most at this probability threshold for polyarteritis nodosa, with 15 out of 18 (83%) cases detected (a 4.3-fold increase in identified cases). The prevalence of autoimmune conditions rose with increasing probabilities from a mean of 1.0% among individuals with a probability less than 0.1 to 3.9% among individuals with a probability equal to 1 (Fig. 3b).

### Prediction of need for future autoantibody testing and rheumatologist referral

**Fig. 4:**
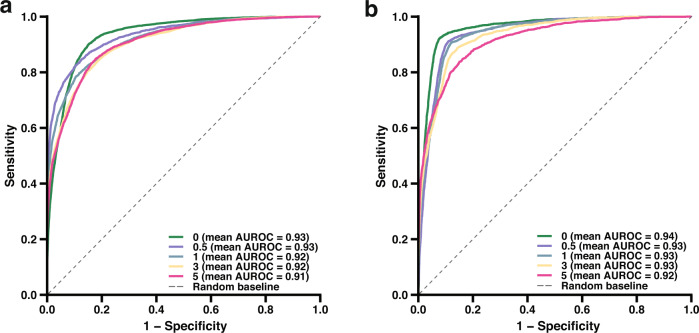
Prediction of future autoantibody and rheumatological testing. **a**, **b** AUROCs of models to predict autoantibody testing and rheumatologist encounter up to 5 years in advance of testing or encounter date, respectively. EHR data were restricted to 0, 0.5, 1, 3, and 5 years prior to the testing or encounter date for participants who had autoantibody testing or a rheumatologist encounter, respectively.

The final objective of the study was to determine the possibility of using the model for prescreening of individuals who need autoantibody testing and rheumatology referrals in advance of their SARDs assessment date (Methods). Using 35,901 individuals from Bio*Me* cohorts 1 and 2, we trained and assessed five models in which EHR data was restricted to 0.5, 1, 3, and 5 years prior to the first date of the autoantibody testing. Individuals in need of autoantibody testing were successfully identified up to 5 years earlier by the models, with AUROC ranging from 0.91–0.93 and accuracy ranging from 0.86–0.89 (Fig. 4a and Supplementary Table 7). Analogously, five models were developed and evaluated with EHR data restricted to 0.5, 1, 3, and 5 years prior to the first date of an encounter with a rheumatologist. The models demonstrated strong predictive performance with AUROC ranging from 0.92–0.94 and accuracy ranging from 0.85–0.93 (Fig. 4b and Supplementary Table 5). These results indicate that symptoms and findings suspicious for SARDs present years earlier than when they are tested^22,23,34^, and that a predictive model can accurately prescreen these individuals to receive timely diagnostic assessment.

## Discussion

SARDs encompass a diverse set of conditions each with distinct phenotypic manifestations; yet, they collectively share patterns of immune-driven symptoms and findings^42–45^ that we hypothesized can be identified in EHR data. Machine learning can analyze vast amounts of complex clinical data, but its clinical translation to rheumatology is in a nascent stage^46^. In the present proof-of-concept study, we used machine learning trained on EHRs to quantitatively and systematically capture clinical suspicion of SARDs, which is the main indication for autoantibody testing and rheumatology referral^17–21^. Importantly, the model was validated on a diverse, real-world population of individuals in three cohorts from two institutions.

Autoantibody testing and evaluation by rheumatologists are critical for the proper diagnosis and clinical care of SARDs; however, their use and access are often limited and delayed^22,23,47,48^. One potential solution presented here is to use a data-driven model to prescreen individuals who need autoantibody testing and rheumatological consultation, prioritizing those with clinical suspicion for SARDs. This precision medicine approach would facilitate judicious use of autoantibody testing in certain individuals, in agreement with Choosing Wisely recommendations^49^. Different time windows of data to predict future need for testing were evaluated, namely all past medical records in the primary analysis and 1-year windows in sensitivity analyses, and are important to consider for implementation of the model as a clinical tool. Notably, the model demonstrated external validity and portability with good performance in an external cohort from a different institution and an independent non-biobank cohort at Mount Sinai. At increasing probability thresholds, the model revealed up to 86% of individuals with an EHR profile representative of a need for SARDs assessment, but had not received any testing or notation of SARDs in their records. Appropriate and timely testing can lead to an earlier and more accurate diagnosis of SARDs, thereby reducing underdiagnosis and improving care^12–15,47,48^. Furthermore, individuals with clinical suspicion for SARDs and rheumatological diseases had high numbers of clinical encounters, indicating the large impact of these conditions on patients, providers, and the healthcare system in line with previous studies^50,51^. Among individuals who eventually received a serological or rheumatological evaluation, the model predicted the need for their evaluation up to 5 years earlier, thus potentially abbreviating what would otherwise be an extensive diagnostic delay. Translation of this model to clinical settings has the potential to aid the diagnostic workflow of physicians by suggesting rheumatological assessment for individuals and accelerating testing and referrals.

The model increased the yield of individuals with autoantibodies and individuals with clinically diagnosed SARDs. Higher probability thresholds enabled the detection of large proportions of individuals harboring autoantibodies; for example, around 80% for probability ≥0.8. Carriers of autoantibodies corresponding to different SARDs had considerably greater probabilities than controls without any autoantibodies; similarly, probabilities for individuals diagnosed with SARDs greatly exceeded that for controls without SARDs. Consistent performance of the model across a wide array of autoantibodies and diseases underscores a shared phenotypic signature^42–45^ among various SARDs that is detectable by artificial intelligence. This approach also identified a subset of individuals with a high probability but no rheumatological testing or SARDs diagnosis who may be underdiagnosed. Ultimately, the aim of this machine learning strategy is to improve diagnostic outcomes, not solely to increase testing, for more individuals. In this vein, we trained the model using EHRs of individuals who were tested for autoantibodies, a key diagnostic tool, but also tested its generalizability to identify cases of other autoimmune conditions. The model showed consistent performance with 18 diverse autoimmune conditions not included in its training dataset, further supporting the hypothesis that a phenotypic profile of numerous immune-driven traits manifesting over time is a hallmark of autoimmune diseases and that models that can recognize this have extensive applicability.

A major objective of the study was to augment the detection of individuals with SARDs. In all datasets, a large share of participants who had autoantibody tests or encounters with rheumatologists had a SARDs diagnosis. It was possible to have trained the model on this narrower subgroup of participants; however, we avoided this approach owing to three key considerations. First, we sought to capture clinical suspicion of SARDs, which represents the primary indication for autoantibody testing or rheumatology consultation. Using these outcomes enabled the model to draw on the clinical gestalt of physicians rather than be trained to predict diagnosis codes with inherent biases and misclassifications^46^. Second, this approach allowed the model to be placed within the diagnostic toolkit of physicians instead of simply generating an output of disease case or control that bypasses the diagnostic process. Third, the sample size was sufficiently large to develop an accurate and portable model, whereas restricting to those with certain diagnosis codes would greatly reduce size and power. Building a model to predict the probability of SARDs itself demands more specialized rheumatological evaluation of disease in individuals, ideally in a prospective manner, which should be endeavored in future studies.

Challenges remain for deploying machine learning models such as the one presented here into the clinical space. The diagnostic performance of a testing modality depends on the prevalence of the target disease, the characteristics of the population being tested, and the properties of the test itself. The model in this study was trained, validated, and externally tested using a balanced set of autoantibody-tested and untested individuals to ensure adequate learning of tested individuals and reduce bias towards the majority class of untested individuals; however, this may limit the model’s PPV in situations with a low prevalence of autoantibody-tested individuals. Nonetheless, there are numerous clinical tests with high NPV and low PPV: HIV^52,53^ and tuberculin skin^54^ tests reliably rule out infection, the Gail Model^55^ estimates the risk of breast cancer, and computed tomography pulmonary angiography (CTPA) aids in the diagnosis of pulmonary embolisms^56^. While these tests produce false positive results (PPV as low as 2–4% in The Gail Model^55^ and 9–15% in CTPA^56^), they are valuable for screening and excluding harmful and burdensome diseases. As this was a proof-of-concept study for detecting and analyzing difficult autoimmune diseases, future studies are needed to evaluate and optimize deployability in different populations with varying prevalences of the disease.

There were several limitations to the study. First, machine learning methods including random forest may be sensitive to overfitting; however, we observed minimal evidence of overfitting as the validation and external test datasets had similar high AUROC. Second, participants were restricted to ≥20 years of age with ≥1 year of EHR data to ensure adequate longitudinal data for the model, which prevented analysis of autoimmune conditions with an earlier age of onset. Third, diagnosis codes were parsed from EHRs and misclassification of codes is possible^57^. Fourth, while the generalizability of the model was assessed with 18 autoimmune conditions not included in the SARDs training dataset, the model was better able to capture cases of conditions with SARDs-like systemic or pain-predominant features (e.g., polyarteritis nodosa and sarcoidosis) as opposed to less similar conditions (e.g., vitiligo). Fifth, the study was retrospective and opportunistic in nature, examining existing EHR data from two biobanks. This led to imbalanced counts of cases and controls, with greater numbers of controls. We mitigated bias due to this imbalance by selecting equal numbers of cases and controls in the training and testing of the model. Prospective studies are needed to further validate the utility of the model to guide changes in clinical care and outcomes of patients.

In summary, we provide an innovative machine learning framework to sift through large-scale multimodal data contained in the EHRs of health systems to identify individuals who should receive serological testing and rheumatologist evaluation, premised on an important hypothesis that presentation of immune-driven phenotypes over time is characteristic of SARDs. We demonstrate that the model can predict the need for different modalities of rheumatological testing, both serological autoantibody tests and rheumatologist consultation, with consistent performance across different datasets and institutions. The model not only stratifies the risk of autoimmune conditions, but also provides an unprecedented opportunity to accelerate and systematize diagnostic testing of SARDs that are often missed or delayed in patients.

## Methods

### Study design and population

We conducted a study to train, validate, and externally test a machine learning model predictive of rheumatic disease testing using clinical features extracted from the EHR of three cohorts across two institutions (Fig. 1a). The model was adapted from a previous model^32^ that predicted CAD risk using EHR data. First, we trained and validated the model using 25,062 EHRs from one cohort in the Bio*Me* Biobank, and externally tested the model on 136,522 EHRs from All of Us. Second, we applied the model to clinical outcomes of prediction of rheumatology encounters, detection of autoantibodies and autoimmune conditions, and prediction of future rheumatological testing in 35,901 participants from two cohorts in the Bio*Me* Biobank. The study protocols were approved by the Institutional Review Board at the Icahn School of Medicine at Mount Sinai (GCO#07-0529; STUDY-11-01139) and informed consent was obtained for all participants. Analyses of All of Us were completed according to the All of Us Code of Conduct and all participants provided informed consent; reported results comply with the All of Us Data and Statistics Dissemination Policy and are presented in groups of at least 20 individuals. The study adhered to the principles of the Declaration of Helsinki.

The Bio*Me* Biobank comprises a longitudinal cohort of over 65,000 individuals of African, European, Hispanic, and other self-reported ethnicities recruited from outpatient centers in the Mount Sinai Health System across New York City from 2007 onwards^58^, with follow-up until 2019. Participants are representative of the communities served and are unselected for particular traits or diseases. All individuals consented to provide biological and DNA samples linked to de-identified EHRs, which contain clinical, laboratory, and demographic information. Participants at least 20 years of age with at least 1 year of EHR data and three documented clinical encounters were selected to ensure cases and controls had sufficient EHR data^59,60^ for training and evaluating the model (Supplementary Fig. 8). The model was subsequently externally tested in All of Us, a prospective cohort of over 490,000 participants as of May 2022 of diverse self-reported ethnicities who were enrolled at participating healthcare sites across the United States from 2017 onwards^61^. Individuals provided informed consent including for sharing EHRs, completed health questionnaires, and underwent a physical exam and biospecimen collection upon enrollment. Participants were selected with the same criteria as in the Bio*Me* Biobank (Supplementary Fig. 8).

### Electronic health record (EHR) data sources

De-identified EHR data were analyzed from the Bio*Me* Biobank and All of Us. The Bio*Me* Biobank sources its data from the Mount Sinai Data Warehouse (MSDW), which uses the Observational Health Data Sciences and Informatics (OHDSI) collaborative’s Observational Medical Outcomes Partnership (OMOP) Common Data Model (CDM). OMOP CDM provides a standardized structure for observational data, common representation of terminologies, vocabularies, coding schemes, and standard analytic routines^1^. For each domain of OMOP CDM, non-standard vocabularies are mapped to standard vocabularies (e.g., International Classification of Diseases version 10 Clinical Modification [ICD-10-CM] mapped to SNOMED-CT in the Condition domain). The standard vocabularies include SNOMED-CT for the Condition domain, RxNorm for the Drug domain, and LOINC for the Measurement domain. Clinical data are extracted from Mount Sinai’s Epic Caboodle database, transformed to the OMOP CDM format, and loaded to the MSDW database with refreshes occurring daily. Further information about MSDW and its data sources can be found at https://labs.icahn.mssm.edu/msdw/data-sources/. All of Us also uses the OMOP CDM structure for its participants’ EHR data, including SNOMED-CT conditions, RxNorm drugs, and LOINC measurements. The consistent ontologies and data schemes across Bio*Me* Biobank and All of Us enabled the direct application of the machine learning model derived in Bio*Me* Biobank to All of Us. Further information about All of Us and its data sources can be accessed at https://www.researchallofus.org/data-tools/methods/.

### Identification of individuals with autoantibody tests and rheumatology encounters

We identified participants who had received at least one of two modalities of rheumatological assessment: autoantibody testing and rheumatology encounter. First, we mined EHRs for the presence of serological testing of autoantibodies corresponding to one of 11 different SARDs (Supplementary Table 1). Tested individuals had at least one autoantibody test and non-tested controls did not have any autoantibody tests. We included tests for autoantibodies with high specificity for a particular SARD^2,17–19,39^, while excluding tests for autoantibodies with low specificity, such as anti-nuclear antibody^40^. Results for tests were noted as negative or positive in the EHR, with the latter result used to identify individuals carrying autoantibodies. Second, we searched EHR encounters and medication datasets for the presence of a rheumatology encounter, defined as documentation in clinical notes of being seen by a rheumatologist (“consult to rheumatology” in the encounters dataset) or treated by a rheumatologist (“per rheumatology” order in the medications dataset). Controls were defined as having no evidence of a rheumatology encounter and no clinical suspicion of SARDs noted in their EHR encounters or past medical history (e.g., mentions of autoantibody tests, rheumatic diseases, or autoimmune diseases).

### Clinical features from the EHR included in the model

Both categorical and continuous data from the EHR were used as clinical features for the model in the Bio*Me* Biobank. Only clinical feature data before the date of the first instance of autoantibody testing were used for cases of autoantibody tests and before the date of the first instance of a rheumatology encounter for cases of rheumatology encounters. Age was defined by the date of the most recent entry of included clinical feature data. Categorical features were derived from a total of 14,695 unique ICD-10 diagnosis codes (ICD-9 codes were converted to ICD-10 in 2016) and 27,802 medications in the EHR, and were coded as presence or absence of the feature. Diagnosis codes corresponding to SARDs (Supplementary Table 1) and common SARD medications (hydroxychloroquine, methotrexate, and azathioprine) were removed to prevent data leakage and circularity in the model. Diagnosed cases of SARDs had at least one corresponding diagnosis code, while controls did not have any corresponding diagnosis codes. Continuous features included 105 laboratory measurements and 9 vital traits. Continuous features with >60% missing values were removed and participants missing >60% of the remaining continuous features were excluded as quality control for accurate imputation. The removed participants had a short duration in the biobank (median, 3.9 years [IQR, 7.3]) and few clinical encounters (median, 6 [IQR, 11]); their median age was 49 years (IQR, 40); 41% were males, 33% European, 21% African, 27% Hispanic, and 10% other ethnicities. The remaining values of continuous features were imputed with a random forest-based algorithm via missForest (version 1.4)^62^. Multiple entries were collapsed as the median value for each participant. Highly correlated continuous features (Pearson’s correlation coefficient >0.90) were removed; the feature with the highest overall correlation to all features was discarded whenever two features were highly correlated. After feature selection (see next subsection, “Building and evaluating the models”), 22 ICD-10 diagnosis codes, 37 medications, 61 laboratory results, and eight vital traits were used to train the machine learning model (Supplementary Table 9).

We externally tested the model in All of Us using EHR data that were also restricted to entries before the date of the first instance of autoantibody testing. Continuous features and participants with >60% missing values were removed, and the remainder of the values were imputed using the aforementioned random forest-based algorithm.

### Building and evaluating the models

We implemented a random forest-based machine learning system^32,63^ using clinical features contained in the EHR to predict rheumatological testing (Fig. 1a and Supplementary Fig. 9). The workflow was repeated 100 times to reduce sampling bias. A training dataset was generated during each iteration with a random sample of 90% of cases (autoantibody-tested individuals) and an equivalent number of controls (non-autoantibody-tested individuals). A balanced validation dataset included the remaining 10% of cases and an equivalent number of controls. Feature selection was completed on the training dataset with the *Boruta* function from the Boruta package (version 7.0.0)^64^ and applied to the validation dataset to decrease model complexity^64^ and increase clinical interpretability of the prediction task^27^. Features not selected were discarded from the validation dataset accordingly. Age, sex, and self-reported ethnicity were included in the model as covariates. Continuous features were scaled and centered in the training dataset; these metrics were then applied to the validation dataset. A tenfold cross-validation scheme was employed in the training dataset to optimize the model’s hyperparameters. The resultant model predicted autoantibody testing status and generated probabilities in the entire population except individuals used in the training dataset and performance metrics were presented as the mean and 95% CI across the 100 iterations. Using this workflow, we subsequently trained 100 new models to predict autoantibody testing in an external test dataset from All of Us. Selected features from the Bio*Me* Biobank that were present in All of Us were used to train a new model for each iteration. Each time, the model predicted autoantibody testing status using all cases and an equal number of randomly sampled controls. The resulting performance metrics were presented as the mean and 95% CI across all 100 iterations. The same workflow was used to develop and validate a model that predicted rheumatologist encounters using EHR clinical features in Bio*Me* Biobank.

We performed several sensitivity analyses. We evaluated the model in a cohort design to guard against temporal bias^65^, in which participant records for a given year were analyzed by the model to predict autoantibody testing in the following year; the model used rolled-up features of diagnosis codes (e.g., M19 feature contains any sublevels such as M19.0, M19.01, M19.011, etc.) and medications (e.g., acetaminophen feature contains acetaminophen of different dosages). We tested the model in a non-biobank cohort of 839,188 participants from the Mount Sinai health system found in MSDW^66^. We also tested the model in a subset of individuals less than or equal to 50 years old, as this group has a higher prevalence of SARDs. We further assessed the model in subgroups stratified by sex, ethnicity, and highest education level (advanced degree/post-college, college, high school, and middle/elementary school).

### Assessment of diverse autoimmune conditions

Numerous autoimmune conditions were not part of the training set because they are not SARDs, or autoantibody testing is not appropriate or informative for their diagnosis. To further validate the model’s ability to detect autoimmune phenotypic signatures, we assessed its performance in identifying individuals diagnosed with a diverse set of 18 autoimmune conditions not included in the original training set: Addison’s disease, ankylosing spondylitis, autoimmune hepatitis, Crohn’s disease, giant cell arteritis, glomerulonephritis, Graves’ disease, Hashimoto’s thyroiditis, multiple sclerosis, myasthenia gravis, optic neuritis, polyarteritis nodosa, psoriasis, rheumatic mitral valve disease, sarcoidosis, type 1 diabetes, ulcerative colitis, and vitiligo (Supplementary Table 10). These were selected because of their diverse representation of body systems and prevalence in the health system, and traits that can be analyzed in the EHR.

### Prediction of future autoantibody tests and rheumatology encounters

We assessed the model’s ability to prescreen individuals in need of rheumatological evaluation in the future, which could prioritize those with clinical suspicion of SARDs for testing and potentially streamline their care. Using 35,901 individuals from Bio*Me* cohorts 1 and 2, we restricted the EHR data of cases—those who had autoantibody testing or rheumatology encounter—to 0, 0.5, 1, 3, and 5 years before the date of the first instance of autoantibody testing or encounter with rheumatologists, respectively. All EHR data were included for controls. These temporally restricted datasets were supplied as inputs to the same models and subjected to the same training and validation workflow as in the primary analysis. For each temporally restricted model, performance metrics were reported as the mean and 95% CI across all 100 iterations of the workflow.

### Statistical analysis

Differences in categorical variables were evaluated with a two-sided unpaired Fisher’s exact test and continuous variables were assessed with Welch’s *t*-test and Mann–Whitney *U*-test. Models to predict autoantibody testing and rheumatology encounters were assessed with AUROC, sensitivity, specificity, accuracy, PPV, NPV, and F1 score using the pROC package (version 1.16.2)^67^. Linear and logistic regression were used to test the association of model probabilities with continuous and categorical outcomes, respectively. Regression models were adjusted for age (defined at the date of last encounter), sex, body mass index, and self-reported ethnicity, unless otherwise stated. The significance level was set at 0.05. All statistical tests and plots were generated with R (version 3.5.3).

### Reporting summary

Further information on research design is available in the Nature Portfolio Reporting Summary linked to this article.

## Supplementary information

Supplementary Information

Reporting Summary

## Data Availability

Data from All of Us is available via application to the Researcher Workbench at https://workbench.researchallofus.org/login. Further information regarding the Bio*Me* Biobank and its dataset are available at https://icahn.mssm.edu/research/ipm/programs/biome-biobank, and further information regarding the Mount Sinai Data Warehouse and its dataset are available at https://labs.icahn.mssm.edu/msdw/data-sources. Access to these data needs to be requested from the Bio*Me* Biobank and Mount Sinai Data Warehouse. Source data are provided with this paper.

## Source data

Source Data

## Code availability

Code for running and analyzing the machine learning model is available at https://data.mendeley.com/datasets/chg348gtxp/1. All plots and statistical tests were generated with R (version 3.5.3). Plots were produced using the pROC (version 1.16.2) and ggplot2 (version 3.3.3) packages, missing values were imputed via a random forest-based algorithm using the missForest (version 1.4) package, features were selected with the *Boruta* function from the Boruta package (version 7.0.0), and the machine learning model was trained and tested using the caret (version 6.0.84) and randomForest (version 4.6–14) packages.

## References

[1] Haag, H., Liang, T., Avina-Zubieta, J. A. & De Vera, M. A. How do patients with systemic autoimmune rheumatic disease perceive the use of their medications: a systematic review and thematic synthesis of qualitative research. *BMC Rheumatol*. **2**, 9 (2018). 10.1186/s41927-018-0017-8 PMC6390776 30886960

[2] Meroni PL (2013). Standardization of autoantibody testing: a paradigm for serology in rheumatic diseases. Nat. Rev. Rheumatol..

[3] Dinse GE (2020). Increasing prevalence of antinuclear antibodies in the United States. Arthritis Rheumatol..

[4] Rees F (2016). The incidence and prevalence of systemic lupus erythematosus in the UK, 1999–2012. Ann. Rheum. Dis..

[5] Carter EE, Barr SG, Clarke AE (2016). The global burden of SLE: prevalence, health disparities and socioeconomic impact. Nat. Rev. Rheumatol..

[6] Kim H (2020). An increased disease burden of autoimmune inflammatory rheumatic diseases in Korea. Semin. Arthritis Rheum..

[7] Kawalec PP, Malinowski KP (2015). The indirect costs of systemic autoimmune diseases, systemic lupus erythematosus, systemic sclerosis and sarcoidosis: a summary of 2012 real-life data from the Social Insurance Institution in Poland.. Expert. Rev. Pharmacoecon. Outcomes Res..

[8] Anaya JM (2014). The diagnosis and clinical significance of polyautoimmunity. Autoimmun. Rev..

[9] Wang L, Wang F-S, Gershwin ME (2015). Human autoimmune diseases: a comprehensive update. J. Intern. Med..

[10] Anaya JM (2010). The autoimmune tautology. Arthritis Res. Ther..

[11] Mosca M (2019). Brief report: how do patients with newly diagnosed systemic lupus erythematosus present? a multicenter cohort of early systemic lupus erythematosus to inform the development of new classification criteria. Arthritis Rheumatol..

[12] Sloan, M. et al. Medically explained symptoms: a mixed methods study of diagnostic, symptom and support experiences of patients with lupus and related systemic autoimmune diseases. *Rheumatol. Adv. Pract*. **4**, rkaa006 (2020). 10.1093/rap/rkaa006 PMC7197794 32373774

[13] Johnson AE, Gordon C, Hobbs FDR, Bacon PA (1996). Undiagnosed systemic lupus erythematosus in the community. Lancet.

[14] Wylezinski LS (2019). Illuminating an invisible epidemic: a systemic review of the clinical and economic benefits of early diagnosis and treatment in inflammatory disease and related syndromes. J. Clin. Med..

[15] Kernder A (2021). Delayed diagnosis adversely affects outcome in systemic lupus erythematosus: cross sectional analysis of the LuLa cohort. Lupus.

[16] Suurmond J, Diamond B (2015). Autoantibodies in systemic autoimmune diseases: specificity and pathogenicity. J. Clin. Invest..

[17] Xiao ZX, Miller JS, Zheng SG (2021). An updated advance of autoantibodies in autoimmune diseases. Autoimmun. Rev..

[18] Chang PY, Yang CT, Cheng CH, Yu KH (2016). Diagnostic performance of anti-cyclic citrullinated peptide and rheumatoid factor in patients with rheumatoid arthritis. Int. J. Rheum. Dis..

[19] Sauerland U (2005). Clinical utility of the anti-CCP assay: experiences with 700 patients. Ann. N. Y. Acad. Sci..

[20] Ingegnoli F, Castelli R, Gualtierotti R (2013). Rheumatoid factors: clinical applications. Dis. Markers.

[21] Castro C, Gourley M (2010). Diagnostic testing and interpretation of tests for autoimmunity. J. Allergy Clin. Immunol..

[22] Meisters R (2020). EULAR/eumusc.net standards of care for rheumatoid arthritis: cross-sectional analyses of importance, level of implementation and care gaps experienced by patients and rheumatologists across 35 European countries. Ann. Rheum. Dis..

[23] Fitzgerald A (2011). Relative urgency for referral from primary care to rheumatologists: the priority referral score. Arthritis Care Res..

[24] Stafford IS (2020). A systematic review of the applications of artificial intelligence and machine learning in autoimmune diseases. npj Digit. Med..

[25] Adlung L, Cohen Y, Mor U, Elinav E (2021). Machine learning in clinical decision making. Med.

[26] Morley TJ (2021). Phenotypic signatures in clinical data enable systematic identification of patients for genetic testing. Nat. Med..

[27] Rajkomar A, Dean J, Kohane I (2019). Machine learning in medicine. N. Engl. J. Med..

[28] Li L (2015). Identification of type 2 diabetes subgroups through topological analysis of patient similarity. Sci. Transl. Med..

[29] Obermeyer Z, Lee TH (2017). Lost in thought — the limits of the human mind and the future of medicine. N. Engl. J. Med..

[30] Rajkomar A (2018). Scalable and accurate deep learning with electronic health records. npj Digit. Med..

[31] Topol EJ (2019). High-performance medicine: the convergence of human and artificial intelligence. Nat. Med..

[32] Forrest IS (2022). Machine learning-based marker for coronary artery disease: derivation and validation in two longitudinal cohorts. Lancet.

[33] Agrawal S (2021). Selection of 51 predictors from 13,782 candidate multimodal features using machine learning improves coronary artery disease prediction. Patterns.

[34] Goldblatt F, O’Neill SG (2013). Clinical aspects of autoimmune rheumatic diseases. Lancet.

[35] Ghassemi M (2020). A review of challenges and opportunities in machine learning for health. AMIA Jt. Summits Transl. Sci. Proc..

[36] Krause J (2018). Grader variability and the importance of reference standards for evaluating machine learning models for diabetic retinopathy. Ophthalmology.

[37] Loftus TJ (2022). Artificial intelligence-enabled decision support in nephrology. Nat. Rev. Nephrol..

[38] Slack WV, Hicks P, Reed CE, Van Cura LJ (1966). A computer-based medical-history system. N. Engl. J. Med..

[39] Ali Y (2018). Rheumatologic tests: a primer for family physicians. Am. Fam. Physician.

[40] Grygiel-Górniak B, Rogacka N, Puszczewicz M (2018). Antinuclear antibodies in healthy people and non-rheumatic diseases – diagnostic and clinical implications. Reumatologia.

[41] Weiss G, Schett G (2013). Anaemia in inflammatory rheumatic diseases. Nat. Rev. Rheumatol..

[42] Szekanecz Z (2021). Autoinflammation and autoimmunity across rheumatic and musculoskeletal diseases. Nat. Rev. Rheumatol..

[43] Iaccarino L (2013). Overlap connective tissue disease syndromes. Autoimmun. Rev..

[44] Davies K, Dures E, Ng WF (2021). Fatigue in inflammatory rheumatic diseases: current knowledge and areas for future research. Nat. Rev. Rheumatol..

[45] Cutolo M, Smith V (2021). Detection of microvascular changes in systemic sclerosis and other rheumatic diseases. Nat. Rev. Rheumatol..

[46] Kingsmore KM, Puglisi CE, Grammer AC, Lipsky PE (2021). An introduction to machine learning and analysis of its use in rheumatic diseases. Nat. Rev. Rheumatol..

[47] Niemantsverdriet E, Dougados M, Combe B, van der Helm-van Mil AHM (2020). Referring early arthritis patients within 6 weeks versus 12 weeks after symptom onset: an observational cohort study. Lancet Rheumatol..

[48] Kvien TK (2020). Considerations for improving quality of care of patients with rheumatoid arthritis and associated comorbidities. RMD Open.

[49] Yazdany J (2013). Choosing wisely: the American College of Rheumatology’s top 5 list of things physicians and patients should question. Arthritis Care Res..

[50] Samnaliev M (2021). Health-care utilization and costs in adults with systemic lupus erythematosus in the United Kingdom: a real-world observational retrospective cohort analysis. Rheumatol. Adv. Pr..

[51] Roodenrijs NMT (2021). Healthcare utilization and economic burden of difficult-to-treat rheumatoid arthritis: a cost-of-illness study. Rheumatology.

[52] Kim S, Lee JH, Choi JY, Kim JM, Kim HS (2010). False-positive rate of a ‘fourth-generation’ HIV antigen/antibody combination assay in an area of low HIV prevalence. Clin. Vaccin. Immunol..

[53] Antelman, G. et al. Balancing HIV testing efficiency with HIV case identification among children and adolescents (2-19 years) using an HIV risk screening approach in Tanzania. *PLoS ONE* **16**, e0251247 (2021). 10.1371/journal.pone.0251247 PMC8101905 33956881

[54] Zhou G (2020). Interferon-γ release assays or tuberculin skin test for detection and management of latent tuberculosis infection: a systematic review and meta-analysis. Lancet Infect. Dis..

[55] Tice JA (2008). Using clinical factors and mammographic breast density to estimate breast cancer risk: Development and validation of a new predictive model. Ann. Intern. Med..

[56] Doğan H, de Roos A, Geleijins J, Huisman M, Kroft L (2015). The role of computed tomography in the diagnosis of acute and chronic pulmonary embolism. Diagn. Interv. Radiol..

[57] Young JC, Conover MM, Jonsson Funk M (2018). Measurement error and misclassification in electronic medical records: methods to mitigate bias. Curr. Epidemiol. Rep..

[58] Tayo BO (2011). Genetic background of patients from a university medical center in Manhattan: Implications for personalized medicine. PLoS ONE.

[59] Li R, Chen Y, Ritchie MD, Moore JH (2020). Electronic health records and polygenic risk scores for predicting disease risk. Nat. Rev. Genet..

[60] Kirby JC (2016). PheKB: a catalog and workflow for creating electronic phenotype algorithms for transportability. J. Am. Med. Inform. Assoc..

[61] Denny JC (2019). The “All of Us” research program. N. Engl. J. Med..

[62] Stekhoven DJ, Bühlmann P (2012). MissForest—non-parametric missing value imputation for mixed-type data. Bioinformatics.

[63] Liaw A, Wiener M (2002). Classification and regression by randomForest. R. N..

[64] Kursa MB, Rudnicki WR (2010). Feature selection with the Boruta package. J. Stat. Softw..

[65] Yuan W (2021). Temporal bias in case-control design: preventing reliable predictions of the future. Nat. Commun..

[66] Datta, S. et al. FIBER: enabling flexible retrieval of electronic health records data for clinical predictive modeling. *JAMIA Open* **4**, ooab048 (2021). 10.1093/jamiaopen/ooab048 PMC8327378 34350388

[67] Robin X (2011). pROC: an open-source package for R and S+ to analyze and compare ROC curves. BMC Bioinforma..

