# Supplementary material for: A machine learning model identifies patients in need of autoimmune disease testing using electronic health records
Source: Nat Commun. 2023 Apr 25;14:2385. doi: 10.1038/s41467-023-37996-7 (PMC10130143; doi:10.1038/s41467-023-37996-7)
Supplement: Supplementary file 1 — Supplementary Information [file 41467_2023_37996_MOESM1_ESM.pdf]

Supplementary Information

A machine learning model identifies patients in need of autoimmune disease testing using electronic health records

Iain S. Forrest[1,2,3,4], Ben O. Petrazzini[1,4], Áine Duffy[1,4], Joshua K. Park[1,2,4], Anya J. O'Neal[5], Daniel M. Jordan[1,4], Ghislain Rocheleau[1,4], Girish N. Nadkarni[1,3,4,6], Judy H. Cho[1,3,4,6], Ashira Blazer[7], Ron Do*[1,3,4].

1. The Charles Bronfman Institute for Personalized Medicine, Icahn School of Medicine at Mount Sinai, New York, NY, USA
2. Medical Scientist Training Program, Icahn School of Medicine at Mount Sinai, New York, NY, USA
3. The Bio*Me* Phenomics Center, Icahn School of Medicine at Mount Sinai, New York, NY, USA
4. Department of Genetics and Genomic Sciences, Icahn School of Medicine at Mount Sinai, New York, NY, USA
5. Department of Microbiology and Immunology, University of Maryland School of Medicine, Baltimore, MD, USA
6. Department of Medicine, Icahn School of Medicine at Mount Sinai, New York, NY, USA
7. Division of Rheumatology, Hospital for Special Surgery, New York, NY, USA

***Corresponding Author**:
Ron Do, PhD
Annenberg Building, Floor 18 Room 80B
1468 Madison Ave
New York, NY-10029
Phone Number: 212-241-6206 | Fax Number: 212-849-2643
ron.do@mssm.edu

TABLE OF CONTENTS

Supplementary figures

Supplementary Fig. 1. SHapley Additive exPlanations (SHAP) analysis of contribution of top 25 features to model prediction
Supplementary Fig. 2. Cohort design evaluation of model performance using rolled up diagnosis codes and medications in internal validation and external test cohorts
Supplementary Fig. 3. Model performance in predicting autoantibody testing in a non-biobank dataset
Supplementary Fig. 4. Model performance in predicting autoantibody testing in subgroups of individuals less than or equal to 50 years old
Supplementary Fig. 5. Model probabilities in subgroups stratified by sex, ethnicity, and education in the Bio*Me* Biobank and All of Us
Supplementary Fig. 6. Model performance in predicting rheumatology encounters in an independent dataset
Supplementary Fig. 7. Model identification of individuals with autoantibodies and diagnoses for SARDs in the external test dataset
Supplementary Fig. 8. Selection of study participants from the Bio*Me* Biobank, All of Us, and Mount Sinai Data Warehouse (MSDW)
Supplementary Fig. 9. Training, validation, external testing, and holdout set evaluation of machine learning model

Supplementary tables

Supplementary Table 1. International Classification of Diseases-10 (ICD-10) diagnosis codes and autoantibody tests corresponding to systemic autoimmune rheumatic diseases (SARDs)
Supplementary Table 2. Top 25 most important features in machine learning model
Supplementary Table 3. Performance metrics of model evaluated with cohort design using rolled up diagnosis codes and medications
Supplementary Table 4. Summary of study participants with and without autoantibody testing in a non-biobank dataset
Supplementary Table 5. Performance metrics of model in subgroups of individuals less than or equal to 50 years old
Supplementary Table 6. Summary of study participants with and without rheumatology encounters in an independent dataset
Supplementary Table 7. Performance metrics of machine learning models predicting future autoantibody testing
Supplementary Table 8. Performance metrics of machine learning models predicting future encounter with rheumatologist
Supplementary Table 9. Clinical features used to train machine learning model
Supplementary Table 10. Set of 18 autoimmune conditions for validation of model

SUPPLEMENTARY FIGURES

Supplementary Fig. 1. SHapley Additive exPlanations (SHAP) analysis of contribution of top 25 features to model prediction.

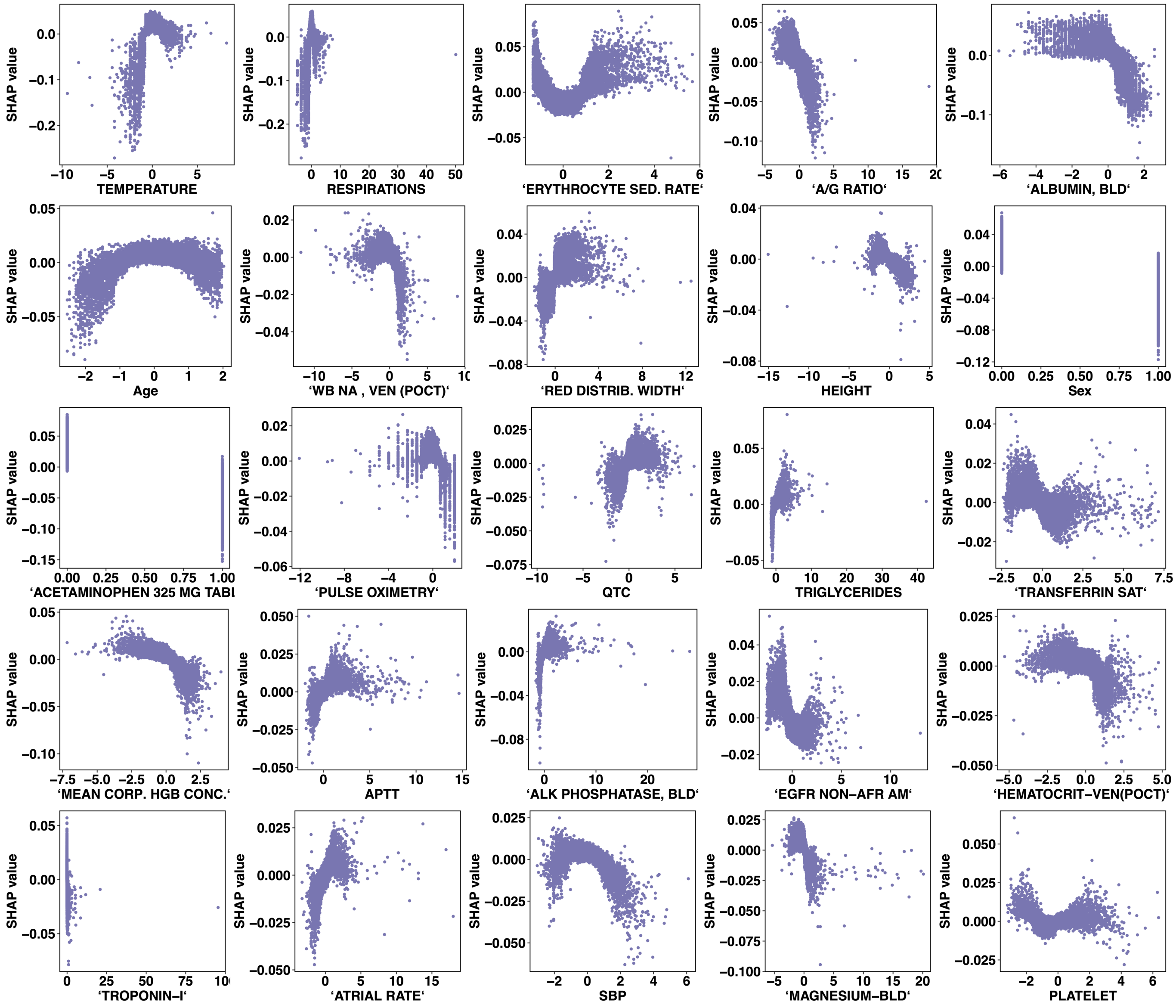

Each point represents one observation; deviation of the value from the mean population value is shown on the X axis. Categorical features are encoded as 1 or 0; continuous features are scaled and centered. A/G, albumin/globulin; APTT, activated partial thromboplastin time; EGFR NON-AFR AM, estimated glomerular filtration rate non-African American; SBP, systolic blood pressure.

Supplementary Fig. 2. Cohort design evaluation of model performance using rolled up diagnosis codes and medications in internal validation and external test cohorts.

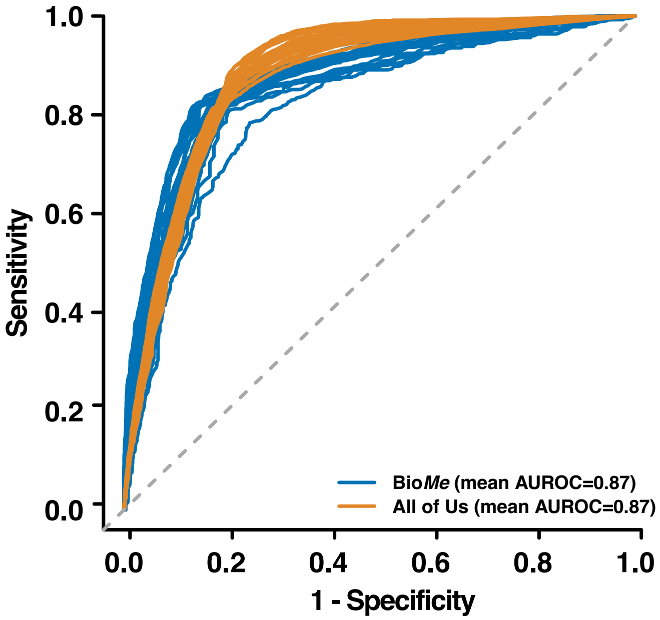

For each given year from 1999-2019, the model was assessed in a cohort design to predict autoantibody testing in the subsequent year. The model used rolled up features of diagnosis codes (e.g., M19 feature contains any sublevels such as M19.0, M19.01, M19.011, etc.) and medications (e.g., acetaminophen feature contains acetaminophen of different dosages). Performance of the model in each year in the internal validation cohort from the Bio*Me* Biobank (Bio*Me*) and the external test cohort from All of Us is depicted as a receiver-operating-characteristic curve, and the mean area under the receiver-operating-characteristic curve (AUROC) across all years is reported in the legend. See Supplementary Table 3 for performance metrics of each model.

Supplementary Fig. 3. Model performance in predicting autoantibody testing in a non-biobank dataset.

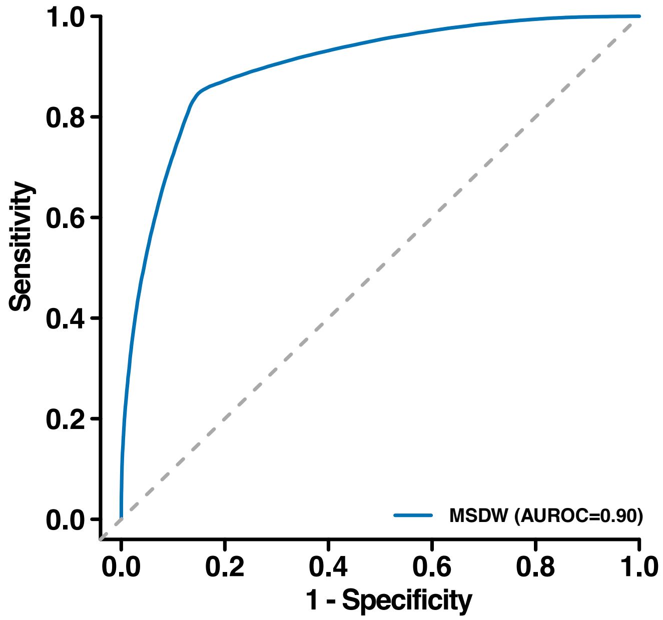

The model was evaluated in a non-biobank cohort of 839,188 participants (67,565 [8.1%] with autoantibody tests) from the Mount Sinai health system found in the Mount Sinai Data Warehouse (MSDW) who had a median of 26 encounters (IQR, 44). In the MSDW dataset, the model had an AUROC of 0.90 (95% CI, 0.89-0.90), accuracy of 0.86 (95% CI, 0.85-0.86), sensitivity of 0.85 (0.85-0.86), specificity of 0.86 (0.86-0.86), negative predictive value of 0.85 (95% CI, 0.85-0.86), and positive predictive value of 0.86 (95% CI, 0.86-0.86).

Supplementary Fig. 4. Model performance in predicting autoantibody testing in subgroups of individuals less than or equal to 50 years old.

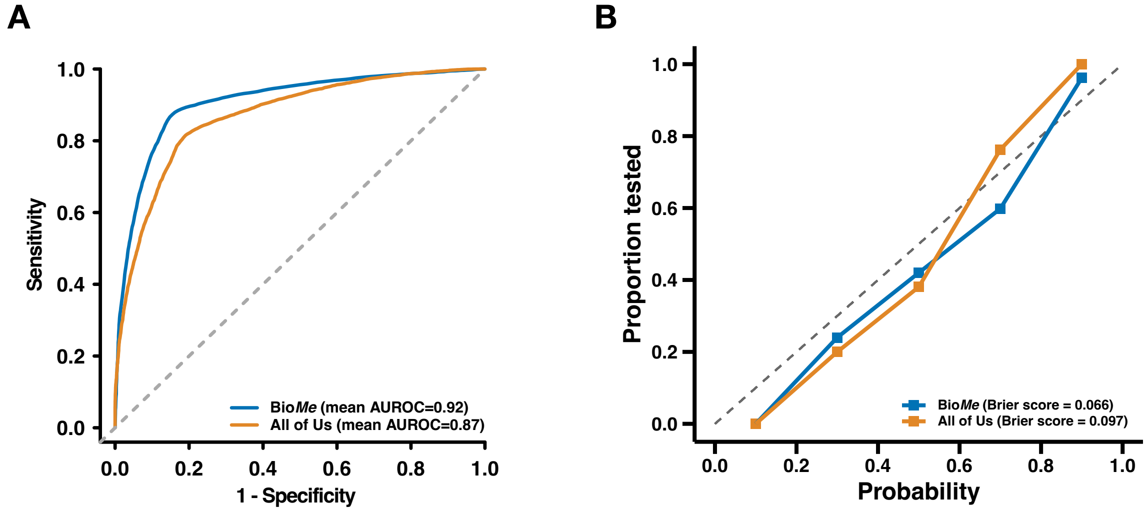

a-b, Performance metrics in a subgroup of individuals less than or equal to 50 years old in the validation dataset from Bio*Me* Biobank (Bio*Me* cohort 1) and the external test dataset from All of Us.

Supplementary Fig. 5. Model probabilities in subgroups stratified by sex, ethnicity, and education in Bio*Me* Biobank and All of Us.

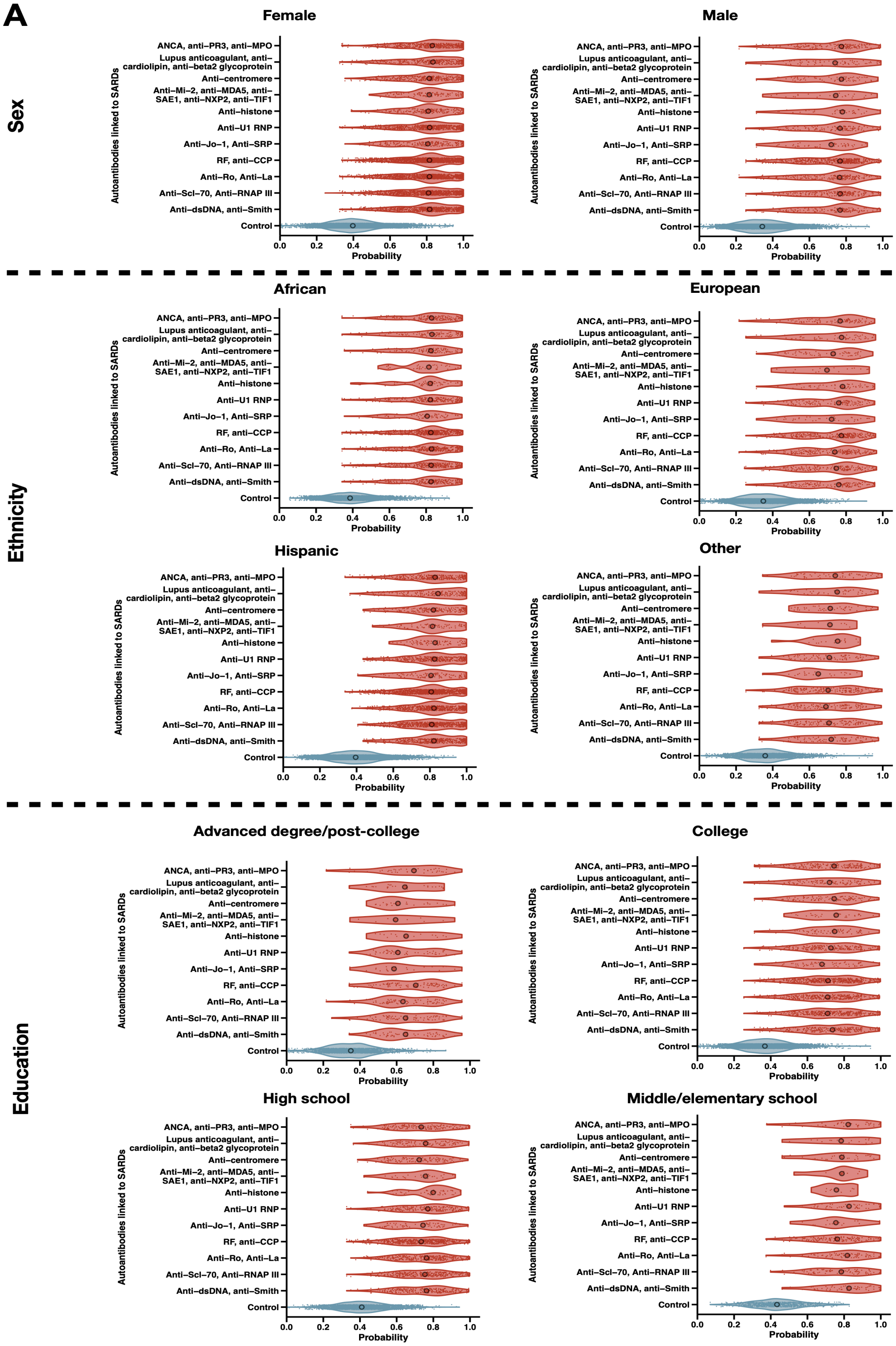

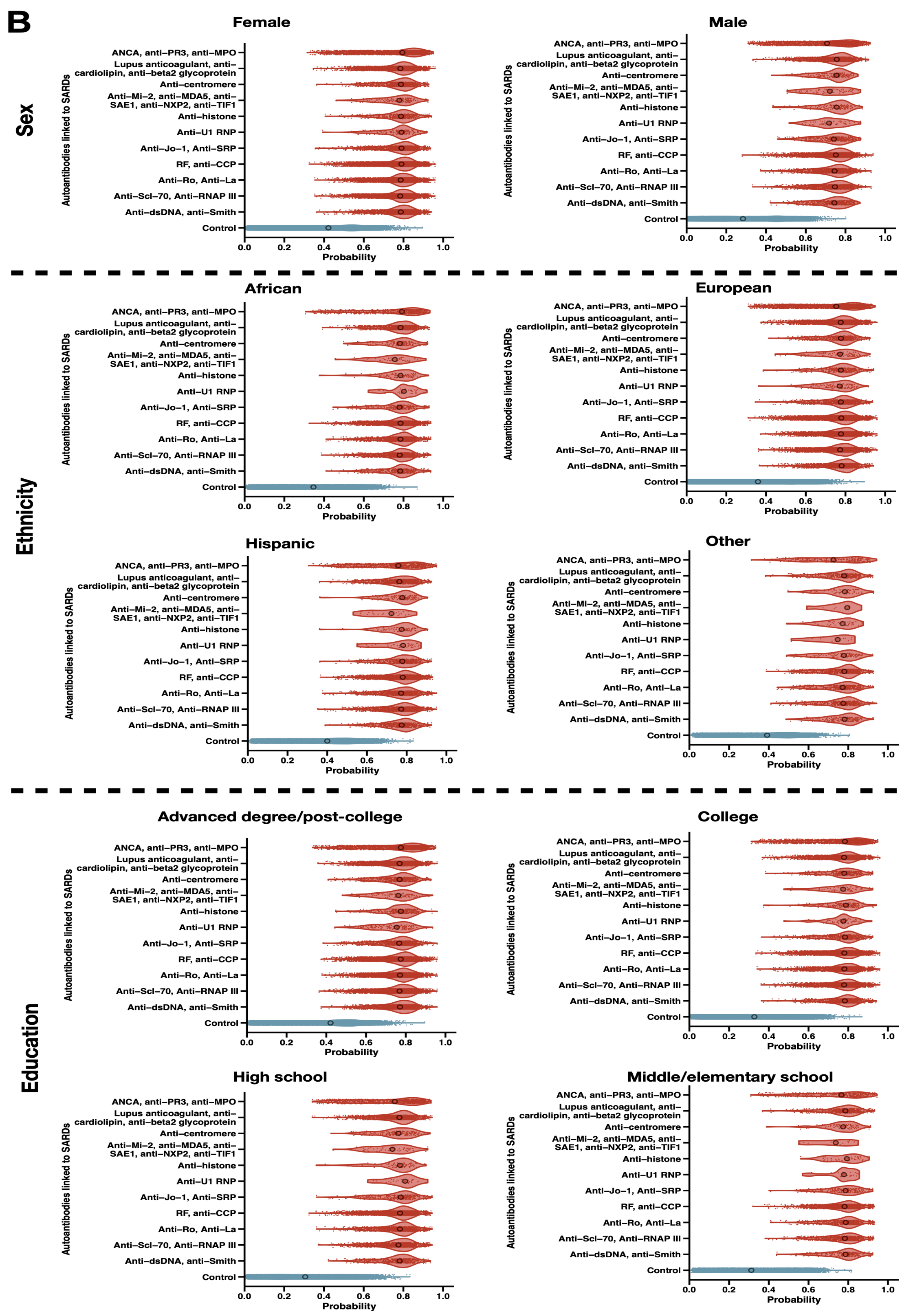

a, Bio*Me* Biobank model-derived probabilities of autoantibody testing for 2,748 participants who had autoantibodies corresponding to SARDs and a rheumatology encounter (red violin plots),

and 20,487 controls who were not tested for autoantibodies and did not have a rheumatology encounter (blue violin plots), stratified by sex, ethnicity, and highest education level. **b**, All of Us model-derived probabilities of autoantibody testing for 24,280 participants who had autoantibodies corresponding to SARDs (red violin plots) and 103,652 controls who were not tested for autoantibodies (blue violin plots), stratified by sex, ethnicity, and highest education level.

Supplementary Fig. 6. Model performance in predicting rheumatology encounters in an independent dataset.

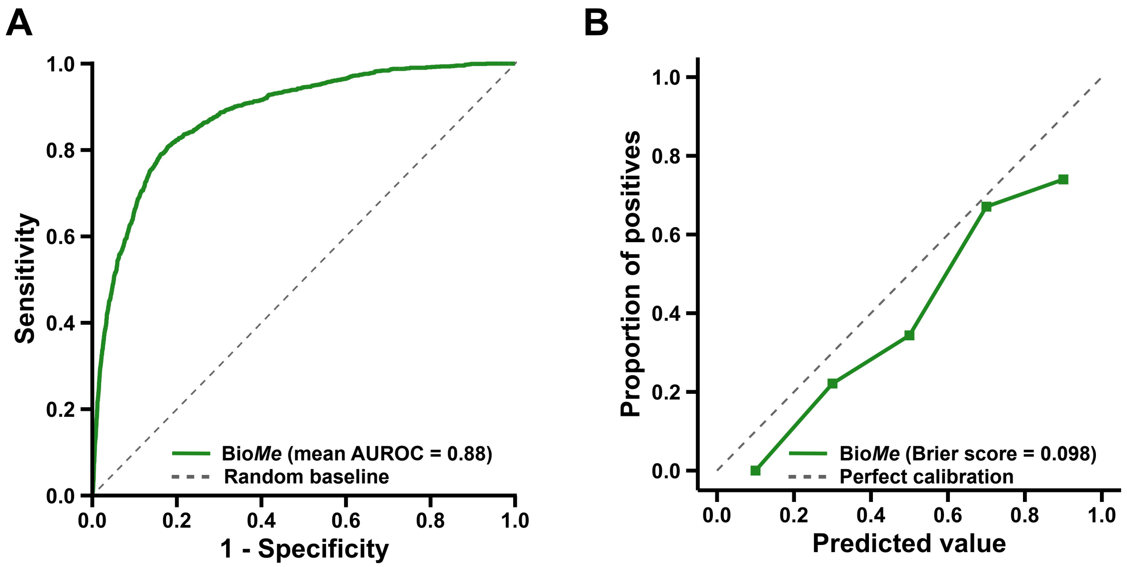

a-c, Performance in an independent dataset from Bio*Me* Biobank (Bio*Me* cohort 2). AUROC, area under the receiver-operating-characteristic curve.

Supplementary Fig. 7. Model identification of individuals with autoantibodies and diagnoses for SARDs in the external test dataset.

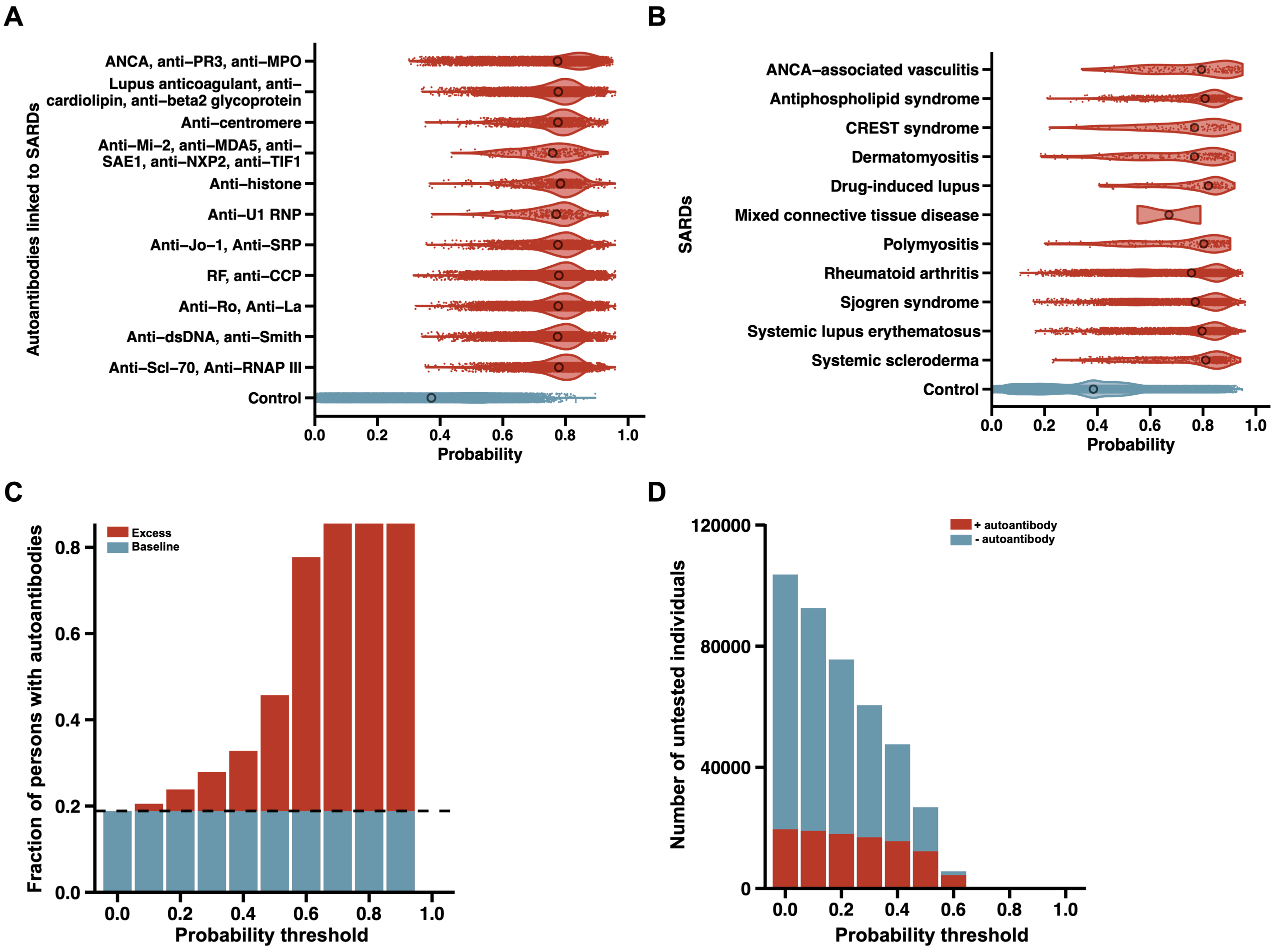

a, Model-derived probabilities of autoantibody testing for 24,280 participants who had autoantibodies corresponding to SARDs (red violin plots) and 103,652 controls who were not tested for autoantibodies (blue violin plot). **b**, Probabilities of autoantibody testing for 9,553 participants with SARDs diagnoses (red violin plots) and 119,223 controls without a SARDs diagnosis or autoantibody test (blue violin plots). **c**, Fraction of individuals with autoantibodies identified by the model at increasing probability thresholds. The dashed line and blue portion of the bar plots represent the baseline fraction of autoantibodies detected in the population (0.19; 24,280 out of 128,775) while the red portion of the bar plots indicate the excess fraction of autoantibodies identified by the model at each probability threshold. **d**, Absolute number of individuals who have not been tested for autoantibodies at increasing probability thresholds; the red portion of the bar plots represents those expected to carry autoantibodies at each probability threshold. At thresholds of ≥0.7 and ≥0.8, 275 out of 288 and 18 out of 19 untested individuals are expected to have autoantibodies, respectively. There were 0 untested individuals at a threshold of 0.9 and no individuals had probability of 1.0.

Supplementary Fig. 8. Selection of study participants from the Bio*Me* Biobank, All of Us, and Mount Sinai Data Warehouse (MSDW).

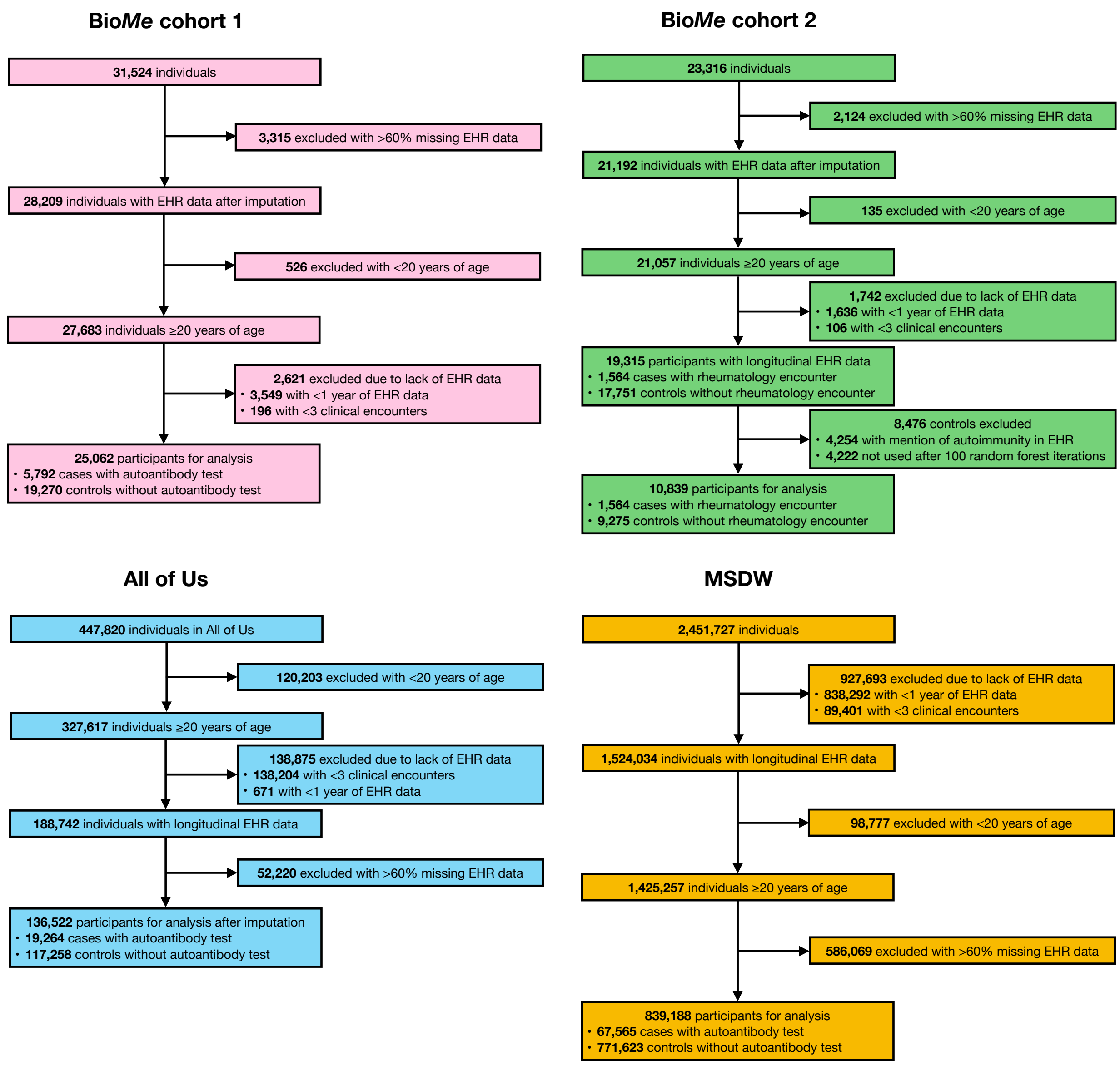

EHR, electronic health record; imputation, random forest-based imputation of continuous laboratory values; rheumatology encounter, individual seen or treated by a rheumatologist; mention of autoimmunity in the EHR, mention of SARDs or autoimmune conditions in clinical notes; not used after 100 random forest iterations, not all controls were included after 100 random forest iterations of a random sample of 90% of cases and an equal number of controls when training and testing the model.

Supplementary Fig. 9. Training, validation, external testing, and holdout set evaluation of machine learning model.

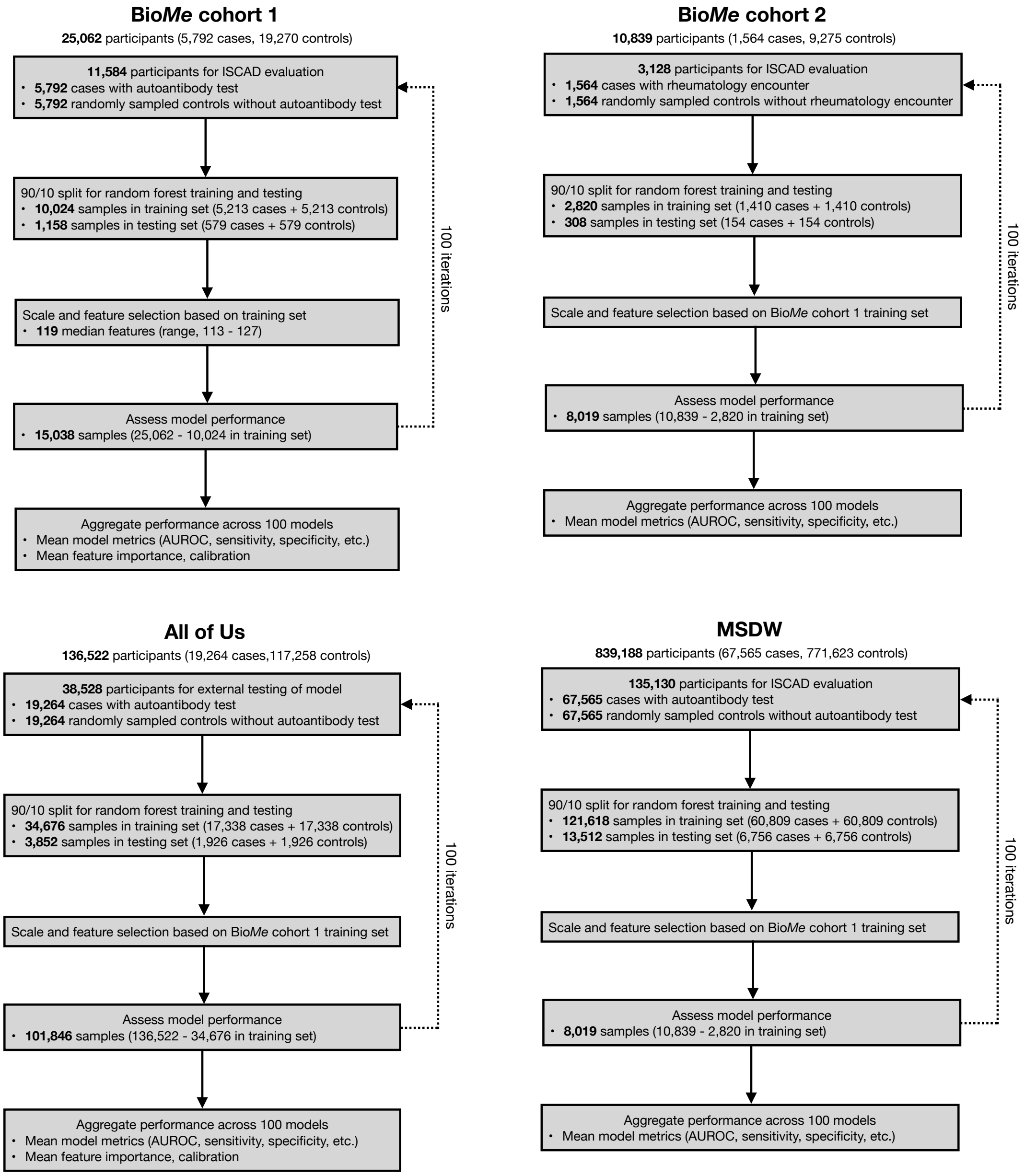

We conducted a study to train, validate, and externally test a machine learning model to predict autoantibody testing using clinical features from the electronic health record (EHR) of participants in two institutions. The model was initially trained and validated in the Bio*Me* Biobank cohort 1, and then externally tested in All of Us. The model was further evaluated for prediction of rheumatology encounters in a holdout set in the Bio*Me* Biobank cohort 2, and prediction of autoantibody testing in a non-biobank population from the Mount Sinai Data Warehouse (MSDW). Features, clinical features in the EHR (diagnosis codes, medications, laboratory measurements, and vitals); AUROC, area under the receiver-operating characteristic curve.

SUPPLEMENTARY TABLES

Supplementary Table 1. International Classification of Diseases-10 (ICD-10) diagnosis codes and autoantibody tests corresponding to systemic autoimmune rheumatic diseases (SARDs).

**SARDs**	**ICD-10**	**Autoantibody**
ANCA-associated vasculitis	M30.1|M31.3|M31.7|I77.82	ANCA, anti-PR3, anti-MPO
Antiphospholipid syndrome	D68.61|D68.62	Lupus anticoagulant, anti-cardiolipin, anti-β2 glycoprotein
Dermatomyositis	M33.0|M33.1|M33.9	Anti-Mi-2, anti-MDA5, anti-SAE1, anti-NXP2, anti-TIF1
Diffuse cutaneous systemic sclerosis	M34*	Anti-Scl-70, Anti-RNAP III
Drug-induced lupus	M32.0	Anti-histone
Limited cutaneous systemic sclerosis	M34.1	Anti-centromere
Mixed connective tissue disease	M35.1|M35.8|M35.9	Anti-U1 RNP
Polymyositis	M33.2|M33.9	Anti-Jo-1, Anti-SRP
Rheumatoid arthritis	M05*|M06*|M08*	RF, anti-CCP
Sjogren syndrome	M35.0*	Anti-Ro**, Anti-La
Systemic lupus erythematosus	L93*|M32.1*|M32.8|M32.9||H01.12	Anti-dsDNA, anti-Smith

, indicates any ICD-10 diagnosis code below the stated parent level (e.g., M34 includes M34.0, M34.1, M34.2, M34.8, and M34.9).
**, refers specifically to anti-Ro60.

Supplementary Table 2. Top 25 most important features in machine learning model.

Feature	Importance
Temperature	93
Respirations	84
Erythrocyte sedimentation rate - Westergren	82
A/G ratio	67
Albumin, blood	64
Age	63
White blood cell count	59
Red cell distribution width	59
Neutrophil #	58
Sex	57
Acetaminophen 325 mg tablet	55
Vitamin D, 25 hydroxy	54
QTc	54
Triglycerides	53
Transferrin saturation	52
Mean corpuscular hemoglobin concentration	52
aPTT	51
Alkaline phosphatase, blood	51
EGFR non-African American	51
Neutrophil %	50
Troponin-I	50
Atrial rate	50
Systolic blood pressure	50
Magnesium, blood	50
Platelet	49

Feature importance was determined for each feature as the mean weight in training the model across 100 iterations, calculated as the feature's mean percent increase in mean squared error divided by its standard deviation and scaled from 0 (least important) to 100 (most important). Further details and units of measurements for features are shown in **Supplementary Table 9**.

Supplementary Table 3. Performance metrics of model evaluated with cohort design using rolled up diagnosis codes and medications.

Dataset	Year	Total n	Autoantibody tested, n (%)	AUROC (95% CI)	Sensitivity (95% CI)	Specificity (95% CI)	Accuracy (95% CI)	NPV (95% CI)	PPV (95% CI)
Bio*Me*	2019	415	28 (6.75)	0.86 (0.81 - 0.88)	0.87 (0.84 - 0.9)	0.87 (0.83 - 0.9)	0.87 (0.84 - 0.89)	0.87 (0.84 - 0.9)	0.87 (0.84 - 0.9)
Bio*Me*	2018	470	18 (3.83)	0.85 (0.81 - 0.87)	0.86 (0.82 - 0.89)	0.86 (0.84 - 0.88)	0.86 (0.84 - 0.88)	0.86 (0.83 - 0.89)	0.86 (0.84 - 0.88)
Bio*Me*	2017	764	33 (4.32)	0.86 (0.82 - 0.88)	0.84 (0.82 - 0.86)	0.85 (0.84 - 0.87)	0.85 (0.83 - 0.86)	0.84 (0.82 - 0.86)	0.85 (0.83 - 0.86)
Bio*Me*	2016	886	42 (4.74)	0.82 (0.79 - 0.84)	0.83 (0.81 - 0.85)	0.82 (0.8 - 0.84)	0.82 (0.81 - 0.83)	0.83 (0.81 - 0.84)	0.82 (0.81 - 0.84)
Bio*Me*	2015	1209	65 (5.38)	0.86 (0.83 - 0.86)	0.87 (0.85 - 0.89)	0.84 (0.83 - 0.86)	0.86 (0.85 - 0.87)	0.87 (0.85 - 0.89)	0.85 (0.84 - 0.86)
Bio*Me*	2014	1825	169 (9.26)	0.88 (0.85 - 0.88)	0.86 (0.84 - 0.87)	0.84 (0.83 - 0.86)	0.85 (0.84 - 0.86)	0.85 (0.84 - 0.87)	0.85 (0.84 - 0.86)
Bio*Me*	2013	1755	140 (7.98)	0.85 (0.83 - 0.85)	0.84 (0.83 - 0.85)	0.84 (0.83 - 0.86)	0.84 (0.84 - 0.85)	0.84 (0.83 - 0.85)	0.85 (0.83 - 0.86)
Bio*Me*	2012	2782	213 (7.66)	0.9 (0.88 - 0.89)	0.83 (0.82 - 0.84)	0.86 (0.85 - 0.87)	0.84 (0.84 - 0.85)	0.83 (0.83 - 0.84)	0.85 (0.84 - 0.86)
Bio*Me*	2011	3371	242 (7.18)	0.89 (0.87 - 0.89)	0.83 (0.82 - 0.84)	0.87 (0.86 - 0.88)	0.85 (0.84 - 0.85)	0.83 (0.83 - 0.84)	0.86 (0.85 - 0.87)
Bio*Me*	2010	3693	221 (5.98)	0.89 (0.87 - 0.89)	0.84 (0.84 - 0.85)	0.84 (0.84 - 0.85)	0.84 (0.84 - 0.85)	0.84 (0.84 - 0.85)	0.84 (0.84 - 0.85)
Bio*Me*	2009	4630	195 (4.21)	0.89 (0.88 - 0.89)	0.84 (0.83 - 0.85)	0.84 (0.83 - 0.85)	0.84 (0.84 - 0.85)	0.84 (0.84 - 0.85)	0.84 (0.84 - 0.85)
Bio*Me*	2008	3818	258 (6.76)	0.89 (0.88 - 0.89)	0.82 (0.81 - 0.83)	0.87 (0.86 - 0.88)	0.84 (0.84 - 0.85)	0.83 (0.82 - 0.84)	0.86 (0.85 - 0.87)

Bio*Me*	2007	3309	155 (4.68)	0.89 (0.88 - 0.89)	0.83 (0.82 - 0.84)	0.86 (0.86 - 0.87)	0.85 (0.84 - 0.85)	0.84 (0.83 - 0.84)	0.86 (0.85 - 0.87)
Bio*Me*	2006	2541	136 (5.35)	0.88 (0.86 - 0.88)	0.83 (0.82 - 0.84)	0.84 (0.83 - 0.85)	0.84 (0.83 - 0.84)	0.83 (0.83 - 0.84)	0.84 (0.83 - 0.85)
Bio*Me*	2005	2227	132 (5.93)	0.87 (0.85 - 0.87)	0.84 (0.83 - 0.85)	0.82 (0.82 - 0.83)	0.83 (0.82 - 0.84)	0.84 (0.83 - 0.85)	0.83 (0.82 - 0.83)
Bio*Me*	2004	1257	48 (3.82)	0.88 (0.85 - 0.88)	0.83 (0.82 - 0.85)	0.84 (0.82 - 0.85)	0.84 (0.83 - 0.84)	0.84 (0.82 - 0.85)	0.84 (0.83 - 0.85)
Bio*Me*	2003	1106	69 (6.24)	0.87 (0.84 - 0.87)	0.83 (0.82 - 0.85)	0.82 (0.8 - 0.84)	0.83 (0.82 - 0.84)	0.83 (0.82 - 0.84)	0.82 (0.81 - 0.84)
Bio*Me*	2002	1097	59 (5.38)	0.88 (0.85 - 0.88)	0.84 (0.83 - 0.85)	0.84 (0.82 - 0.86)	0.84 (0.83 - 0.85)	0.84 (0.83 - 0.85)	0.84 (0.83 - 0.85)
Bio*Me*	2001	1198	85 (7.1)	0.86 (0.84 - 0.86)	0.82 (0.81 - 0.84)	0.82 (0.8 - 0.83)	0.82 (0.81 - 0.83)	0.82 (0.81 - 0.84)	0.82 (0.81 - 0.83)
Bio*Me*	2000	1586	26 (1.64)	0.86 (0.84 - 0.86)	0.86 (0.85 - 0.87)	0.81 (0.8 - 0.82)	0.83 (0.82 - 0.84)	0.85 (0.84 - 0.86)	0.82 (0.81 - 0.83)
Bio*Me*	1999	1730	16 (0.92)	0.89 (0.87 - 0.89)	0.86 (0.85 - 0.87)	0.83 (0.82 - 0.84)	0.84 (0.83 - 0.85)	0.85 (0.84 - 0.86)	0.83 (0.82 - 0.84)
All of Us	2019	68720	787 (1.15)	0.86 (0.86 - 0.88)	0.8 (0.79 - 0.81)	0.84 (0.83 - 0.85)	0.82 (0.82 - 0.82)	0.81 (0.8 - 0.81)	0.83 (0.83 - 0.84)
All of Us	2018	72691	1193 (1.64)	0.87 (0.87 - 0.88)	0.81 (0.8 - 0.82)	0.84 (0.83 - 0.84)	0.82 (0.82 - 0.83)	0.82 (0.81 - 0.82)	0.83 (0.83 - 0.84)
All of Us	2017	62130	1334 (2.15)	0.86 (0.87 - 0.88)	0.82 (0.81 - 0.83)	0.83 (0.83 - 0.83)	0.82 (0.82 - 0.83)	0.82 (0.81 - 0.83)	0.83 (0.83 - 0.83)
All of Us	2016	53780	1100 (2.05)	0.86 (0.87 - 0.88)	0.83 (0.82 - 0.85)	0.83 (0.82 - 0.83)	0.83 (0.82 - 0.84)	0.83 (0.82 - 0.84)	0.83 (0.82 - 0.83)
All of Us	2015	46059	968 (2.1)	0.86 (0.87 - 0.88)	0.85 (0.84 - 0.87)	0.82 (0.81 - 0.83)	0.83 (0.83 - 0.84)	0.85 (0.84 - 0.86)	0.82 (0.82 - 0.83)
All of Us	2014	40444	755 (1.87)	0.86 (0.87 - 0.88)	0.86 (0.85 - 0.87)	0.81 (0.8 - 0.82)	0.84 (0.83 - 0.84)	0.85 (0.84 - 0.86)	0.82 (0.82 - 0.82)

All of Us	2013	32784	605 (1.85)	0.86 (0.87 - 0.88)	0.84 (0.82 - 0.85)	0.82 (0.81 - 0.83)	0.83 (0.82 - 0.83)	0.83 (0.82 - 0.85)	0.82 (0.82 - 0.83)
All of Us	2012	26613	528 (1.98)	0.86 (0.86 - 0.87)	0.85 (0.84 - 0.86)	0.81 (0.8 - 0.81)	0.83 (0.82 - 0.83)	0.84 (0.83 - 0.85)	0.81 (0.81 - 0.82)
All of Us	2011	21716	475 (2.19)	0.86 (0.87 - 0.88)	0.86 (0.85 - 0.87)	0.81 (0.81 - 0.82)	0.83 (0.83 - 0.84)	0.85 (0.84 - 0.86)	0.82 (0.82 - 0.82)
All of Us	2010	18102	396 (2.19)	0.87 (0.87 - 0.88)	0.87 (0.86 - 0.88)	0.81 (0.8 - 0.81)	0.84 (0.83 - 0.84)	0.86 (0.85 - 0.87)	0.82 (0.81 - 0.82)
All of Us	2009	14095	325 (2.31)	0.87 (0.87 - 0.89)	0.87 (0.86 - 0.87)	0.82 (0.81 - 0.82)	0.84 (0.84 - 0.85)	0.86 (0.85 - 0.87)	0.82 (0.82 - 0.83)
All of Us	2008	13005	299 (2.3)	0.87 (0.87 - 0.89)	0.87 (0.85 - 0.89)	0.81 (0.8 - 0.82)	0.84 (0.83 - 0.85)	0.86 (0.84 - 0.88)	0.82 (0.81 - 0.83)
All of Us	2007	10584	262 (2.48)	0.87 (0.88 - 0.89)	0.88 (0.87 - 0.89)	0.82 (0.81 - 0.82)	0.85 (0.84 - 0.86)	0.87 (0.86 - 0.88)	0.83 (0.82 - 0.83)
All of Us	2006	9530	192 (2.01)	0.87 (0.86 - 0.89)	0.86 (0.85 - 0.88)	0.81 (0.8 - 0.81)	0.84 (0.83 - 0.84)	0.86 (0.84 - 0.87)	0.82 (0.81 - 0.82)
All of Us	2005	7537	156 (2.07)	0.87 (0.87 - 0.88)	0.87 (0.86 - 0.88)	0.81 (0.8 - 0.82)	0.84 (0.83 - 0.85)	0.86 (0.86 - 0.87)	0.82 (0.81 - 0.83)
All of Us	2004	6132	145 (2.36)	0.88 (0.88 - 0.9)	0.9 (0.88 - 0.91)	0.8 (0.79 - 0.82)	0.85 (0.84 - 0.86)	0.89 (0.87 - 0.9)	0.82 (0.81 - 0.83)
All of Us	2003	4952	119 (2.4)	0.88 (0.87 - 0.9)	0.86 (0.84 - 0.87)	0.82 (0.81 - 0.82)	0.84 (0.83 - 0.85)	0.85 (0.83 - 0.87)	0.82 (0.81 - 0.83)
All of Us	2002	3641	98 (2.69)	0.87 (0.87 - 0.89)	0.87 (0.85 - 0.89)	0.82 (0.8 - 0.83)	0.84 (0.83 - 0.86)	0.86 (0.84 - 0.88)	0.82 (0.81 - 0.84)
All of Us	2001	3490	98 (2.81)	0.88 (0.88 - 0.9)	0.88 (0.86 - 0.89)	0.81 (0.8 - 0.82)	0.84 (0.83 - 0.85)	0.87 (0.85 - 0.88)	0.82 (0.81 - 0.83)
All of Us	2000	2806	72 (2.57)	0.88 (0.88 - 0.9)	0.88 (0.86 - 0.91)	0.81 (0.79 - 0.83)	0.85 (0.83 - 0.86)	0.88 (0.85 - 0.9)	0.82 (0.81 - 0.84)
All of Us	1999	2182	50 (2.29)	0.87 (0.87 - 0.9)	0.89 (0.86 - 0.92)	0.8 (0.78 - 0.83)	0.85 (0.83 - 0.86)	0.88 (0.85 - 0.91)	0.82 (0.8 - 0.84)

For each given year from 1999-2019, the model was assessed in a cohort design to predict autoantibody testing in the subsequent year. The model used rolled up features of diagnosis codes (e.g., M19 feature contains any sublevels such as M19.0, M19.01, M19.011, etc.) and medications (e.g., acetaminophen feature contains acetaminophen of different dosages). Performance of the model in each year in the internal validation cohort from the Bio*Me* Biobank (Bio*Me*) and the external test cohort from All of Us is tabulated. Area under the receiver-operating-characteristic curve (AUROC); NPV, negative predictive value; PPV, positive predictive value.

Supplementary Table 4. Summary of study participants with and without autoantibody testing in a non-biobank dataset.

Trait	Autoantibody tested (n=67,565)	Not tested (n=771,623)
Age, median (IQR) years	58 (28)	54 (33)
Male, n (%)	21,054 (31)	325,472 (42)
Ethnicity, n (%)		
African	10,887 (16)	112,914 (15)
European	33,760 (50)	394,815 (51)
Hispanic	18,468 (27)	217,079 (28)
Other	4,548 (6.7)	47,850 (6.2)
Interactions with health system		
Duration, median (IQR) years	6.0 (6.1)	5.0 (5.4)
Encounters, median (IQR)	52 (90)	25 (41)

Non-biobank dataset was from the Mount Sinai Data Warehouse (MSDW). Age, age at last clinical encounter; Ethnicity, self-reported ethnicity; Other, self-reported ethnicity other than the listed ones; Duration, length of electronic health record.

Supplementary Table 5. Performance metrics of model in subgroups of individuals less than or equal to 50 years old.

Dataset	AUROC (95% CI)	Sensitivity (95% CI)	Specificity (95% CI)	Accuracy (95% CI)	NPV (95% CI)	PPV (95% CI)
Bio*Me* Biobank	0.92 (0.91 - 0.92)	0.90 (0.90 - 0.91)	0.86 (0.85 - 0.87)	0.87 (0.86 - 0.87)	0.90 (0.89 - 0.90)	0.88 (0.87 - 0.88)
All of Us	0.87 (0.87 - 0.87)	0.82 (0.81 - 0.82)	0.82 (0.81 - 0.82)	0.82 (0.81 - 0.82)	0.82 (0.82 - 0.82)	0.82 (0.81 - 0.82)

AUROC, area under the receiver-operating-characteristic curve; NPV, negative predictive value; PPV, positive predictive value.

Supplementary Table 6. Summary of study participants with and without rheumatology encounters in an independent dataset.

Trait	Rheumatology encounter (n=1,564)	No rheumatology encounter (n=9,275)
Age, median (IQR) years	60 (21)	55 (29)
Male, n (%)	429 (27)	6044 (45)
Ethnicity, n (%)		
African	297 (19)	2322 (17)
European	277 (18)	4621 (34)
Hispanic	790 (51)	4081 (30)
Other	200 (13)	2474 (18)
Interactions with health system		
Unique ICD-10 codes, median (IQR)	69 (55)	29 (31)
Duration, median (IQR) years	7.3 (4.3)	5.6 (4.7)
Encounters, median (IQR)	87 (90)	32 (45)

Independent dataset was from Bio*Me* cohort 2. Age, age at last clinical encounter; Ethnicity, self-reported ethnicity; Other, self-reported ethnicity other than the listed ones; ICD-10, International Classification of Diseases 10; Duration, length of electronic health record.

Supplementary Table 7. Performance metrics of machine learning models predicting future autoantibody testing.

Time prior to test date (years)	AUROC (95% CI)	Sensitivity (95% CI)	Specificity (95% CI)	Accuracy (95% CI)	NPV (95% CI)	PPV (95% CI)
0	0.93 (0.92 - 0.93)	0.91 (0.90 - 0.91)	0.88 (0.87 - 0.88)	0.89 (0.89 - 0.89)	0.90 (0.90 - 0.91)	0.88 (0.88 - 0.88)
0.5	0.93 (0.93 - 0.94)	0.84 (0.84 - 0.85)	0.87 (0.87 - 0.87)	0.86 (0.85 - 0.86)	0.85 (0.84 - 0.85)	0.87 (0.86 - 0.87)
1	0.92 (0.92 - 0.93)	0.84 (0.84 - 0.85)	0.86 (0.86 - 0.86)	0.85 (0.85 - 0.85)	0.85 (0.84 - 0.85)	0.86 (0.86 - 0.86)
3	0.92 (0.92 - 0.93)	0.85 (0.85 - 0.86)	0.86 (0.85 - 0.86)	0.85 (0.85 - 0.86)	0.85 (0.85 - 0.86)	0.86 (0.85 - 0.86)
5	0.91 (0.91 - 0.91)	0.87 (0.87 - 0.88)	0.84 (0.84 - 0.85)	0.86 (0.86 - 0.86)	0.87 (0.87 - 0.87)	0.85 (0.85 - 0.85)

Time prior to test date (years), number of years that electronic health record data was restricted to prior to first test date; AUROC, area under the receiver-operating-characteristic curve; NPV, negative predictive value; PPV, positive predictive value.

Supplementary Table 8. Performance metrics of machine learning models predicting future encounter with rheumatologist.

Time prior to encounter date (years)	AUROC (95% CI)	Sensitivity (95% CI)	Specificity (95% CI)	Accuracy (95% CI)	NPV (95% CI)	PPV (95% CI)
0	0.94 (0.94 - 0.95)	0.92 (0.92 - 0.92)	0.94 (0.93 - 0.94)	0.93 (0.93- 0.93)	0.92 (0.92 - 0.92)	0.93 (0.93 - 0.94)
0.5	0.93 (0.92 - 0.93)	0.90 (0.90 - 0.91)	0.90 (0.90 - 0.90)	0.90 (0.90 - 0.90)	0.90 (0.90 - 0.91)	0.90 (0.90 - 0.90)
1	0.93 (0.92 - 0.93)	0.91 (0.90 - 0.91)	0.90 (0.90 - 0.90)	0.90 (0.90 - 0.90)	0.91 (0.90 - 0.91)	0.90 (0.90 - 0.90)
3	0.93 (0.92 - 0.93)	0.89 (0.88 - 0.89)	0.89 (0.89 - 0.89)	0.89 (0.89 - 0.90)	0.89 (0.89 - 0.89)	0.89 (0.89 - 0.89)
5	0.92 (0.91 - 0.92)	0.84 (0.84 - 0.84)	0.86 (0.86 - 0.87)	0.85 (0.85 - 0.85)	0.84 (0.84 - 0.85)	0.86 (0.86 - 0.86)

Time prior to test date (years), number of years that electronic health record data was restricted to prior to first encounter date; AUROC, area under the receiver-operating-characteristic curve; NPV, negative predictive value; PPV, positive predictive value.

Supplementary Table 9. Clinical features used to train machine learning model.

Feature	Participants (N=23,938)
Diagnosis codes — no. (%)	
E55.9 - Vitamin D deficiency, unspecified	7328 (29)
E66.3 - Overweight	2756 (11)
E66.9 - Obesity, unspecified	5353 (21)
E78.00 - Pure hypercholesterolemia, unspecified	4096 (16)
E78.5 - Hyperlipidemia, unspecified	8227 (33)
G89.29 - Other chronic pain	4738 (19)
I10 - Essential (primary) hypertension	12146 (49)
I25.10 - Atherosclerotic heart disease of native coronary artery without angina pectoris	3538 (14)
J06.9 - Acute upper respiratory infection, unspecified	4877 (20)
J45.909 - Unspecified asthma, uncomplicated	3933 (16)
K21.9 - Gastro-esophageal reflux disease without esophagitis	6369 (25)
M19.90 - Unspecified osteoarthritis, unspecified site	3018 (12)
R73.03 - Prediabetes	3484 (14)
Z00.00 - Encounter for general adult medical examination without abnormal findings	13471 (54)
Z01.419 - Encounter for gynecological examination (general) (routine) without abnormal findings	5605 (22)
Z01.810 - Encounter for preprocedural cardiovascular examination	2390 (9.5)
Z01.818 - Encounter for other preprocedural examination	5249 (21)
Z11.3 - Encounter for screening for infections with a predominantly sexual mode of transmission	2861 (11)
Z11.59 - Encounter for screening for other viral diseases	2819 (11)
Z12.11 - Encounter for screening for malignant neoplasm of colon	5592 (22)
Z13.220 - Encounter for screening for lipoid disorders	2670 (11)
Z23 - Encounter for immunization	14446 (58)
Medications — no. (%)	
Acetaminophen 325 mg tablet	8815 (35)
Albuterol sulfate hfa 90 mcg/actuation aerosol inhaler	5244 (21)
Amlodipine 10 mg tablet	2558 (10)
Amlodipine 5 mg tablet	3458 (14)
Aspirin 81 mg chewable tablet	5025 (20)
Aspirin 81 mg tablet, delayed release	2741 (11)
Atorvastatin 20 mg tablet	2484 (9.9)
Atorvastatin 40 mg tablet	2704 (11)
Cephalexin 500 mg capsule	2863 (11)
Ciprofloxacin 500 mg tablet	2852 (11)
Dextrose 40 % oral gel	2646 (11)
Dextrose 50 % in water (d50w) intravenous solution	2621 (11)
Docusate sodium 100 mg capsule	6290 (25)
Famotidine 20 mg tablet	3456 (14)
Flu vaccine 2015	3092 (12)
Flu vaccine 2012-13	4571 (18)
Flu vaccine 2016-17	2771 (11)
Flu vaccine 2013-14	2651 (11)
Flu vaccine 2014-15	4003 (16)
Fluticasone 50 mcg/actuation nasal spray, suspension	3860 (15)
Glucagon (human recombinant) 1 mg/ml solution for injection	2727 (11)

Heparin (porcine) 5,000 unit/ml injection solution	3909 (16)
Hydrochlorothiazide 25 mg tablet	2516 (10)
Ibuprofen 400 mg tablet	4943 (20)
Ibuprofen 600 mg tablet	3285 (13)
Ketorolac 30 mg/ml (1 ml) injection solution	2350 (9.4)
Lisinopril 10 mg tablet	2379 (9.5)
Naproxen 500 mg tablet	2972 (12)
Ondansetron HCl (pf) 4 mg/2 ml injection solution	5064 (20)
Oxycodone 5 mg tablet	2869 (11)
Oxycodone-acetaminophen 5 mg-325 mg tablet	7756 (31)
Pantoprazole 40 mg tablet, delayed release	3411 (14)
Pneumococcal 13-val conjugate vaccine	4025 (16)
Polyethylene glycol 3350 17 gram oral powder packet	4575 (18)
Sennosides 8.6 mg tablet	3589 (14)
Sodium chloride 0.9 % intravenous solution	3340 (13)
Sodium chloride 0.9 % iv bolus	7318 (29)
Laboratory measurements — median (IQR)	
A/G Ratio	1.5 (0.37)
Albumin, Blood — g/dL	4.2 (0.5)
Alkaline Phosphatase, Blood — U/L	76 (31)
Amylase, Blood — U/L	71 (23)
APTT — sec	30 (3.2)
Atrial Rate (via electrocardiogram) — min^{-1}	74 (14)
Basophil %	0.40 (0.29)
Bilirubin Direct — mg/dL	0.15 (0.10)
Bilirubin Total — mg/dL	0.40 (0.30)
Calcium, Blood — mg/dL	9.4 (0.59)
Carbon Dioxide-Blood — mEq/L	26 (2.7)
Chloride-Blood — mEq/L	103 (2.5)
Creatinine-Serum — mg/dL	0.90 (0.33)
EGFR Non-African American — $mL/min/1.73m^2$	74 (34)
Ejection Fraction — %	62 (4.2)
Erythrocyte Sedimentation Rate - Westergren — mm/hr	22 (24)
Ferritin — µg/L	98 (118)
Gamma-Glutamyl Transpeptidase-Blood — U/L	33 (30)
Glucose — mg/dL	91 (22)
Glucose (POCT) By Meter — mg/dL	112 (28)
HDL Cholesterol — mg/dL	53 (18)
Hematocrit-Venous (POCT) — %	42 (6)
Hemoglobin A1c — %	5.7 (0.80)
INR	1.0 (0.10)
LDH, blood — mg/dL	
Lipase — U/L	80 (46)
Magnesium, Blood — mEq/L	2.04 (0.17)
Mean Corpuscular Hemoglobin Concentration — g/dL	34 (0.90)
Mean Corpuscular Volume — fL	34 (0.90)
Neutrophil #	4.3 (2.3)
Neutrophil %	62 (13)
Nucleated RBC #	0 (0)

pH - Dipstick	6 (0.65)
Phosphorus-Blood — mg/dL	3.5 (0.32)
Platelet — 10^9/L	231 (81)
PO2, Venous (POCT) — mmHg	32 (9.0)
QRS Duration — ms	85 (10)
QT — ms	389 (32)
QTc — ms	431 (24)
R Axis — °	29 (39)
Red Blood Cell — 10^{12}/L	4.3 (0.67)
Red Cell Distribution Width — %	14 (1.6)
Specific Gravity-Dipstick	1.02 (0.0050)
TIBC — µg/dL	297 (39)
Transferrin Saturation — %	27 (10)
Triglycerides — mg/dL	111 (65)
Troponin-I — ng/dL	0.026 (0.12)
Urine-Creatinine (Concentration) — mg/dL	117 (70)
Urine-pH	6.0 (0.91)
Urine-Specific Gravity	1.02 (0.0080)
Urea Nitrogen-Blood — mg/dL	15 (6.5)
Urobilinogen — mg/dL	0.21 (0.21)
Urobilinogen - Dipstick — mg/dL	0.25 (0.18)
Vitamin D, 25 Hydroxy — ng/mL	25 (9.4)
White Blood Cell — 10^9/L	6.9 (2.7)
Whole Blood Calcium, Venous (POCT) — mg/dL	1.2 (0.032)
Whole Blood Chloride, Venous — mEq/L	103 (2.0)
Whole Blood CO2, Venous — mEq/L	27 (2.9)
Whole Blood Lactate-Venous (POCT) — mEq/L	1.2 (0.48)
Whole Blood Sodium, Venous (POCT) — mEq/L	139 (2.6)
Whole Blood Sodium, Venous — mEq/L	141 (2.0)
Vitals — median (IQR)	
Diastolic Blood Pressure — mmHg	72 (10)
Height — in	65 (5)
Oxygen saturation — % on room air	98 (1.5)
Pain - Abdomen	0 (0)
Pain - Ankle	0 (0)
Pain - Back	0 (0)
Pain - Breast	0 (0)
Pain - Chest	0 (0)
Pain - Left Costal	0 (0)
Pain - Right Costal	0 (0)
Pain - Elbows	0 (0)
Pain - Generalized	0 (0)
Pain - Groin	0 (0)
Pain - Hands	0 (0)
Pain - Head	0 (0)
Pain - Knees	0 (0)
Pain - Left Leg	0 (0)
Pain - Lower Extremities	0 (0)
Pain - Neck	0 (0)

Pain - Pelvis	0 (0)
Pain - Perineum	0 (0)
Pain - Right Leg	0 (0)
Pain - Sacrum	0 (0)
Pain - Scrotum	0 (0)
Pain - Shoulder	0 (0)
Pain - Throat	0 (0)
Pain - Upper Extremities	0 (0)
Pain - Wrist	0 (0)
Pulse (via pulse oximetry) — sec^{-1}	77 (13)
Respirations — min^{-1}	18 (1.0)
Systolic Blood Pressure — mmHg	126 (18)
Temperature — °F	98 (0.60)

IQR, interquartile range; diagnosis codes, International Classification of Diseases (ICD)-10 codes; IV, intravenous; IM, intramuscular; A/G, albumin/globulin; APTT, activated partial thromboplastin time; EGFR, estimated glomerular filtration rate; INR, international normalized ratio; HDL, high-density lipoprotein; TIBC, total iron-binding capacity; pain, pain scale from 0 (no pain) to 10 (worst pain).

Supplementary Table 10. Set of 18 autoimmune conditions for validation of model.

Autoimmune condition	ICD-10 diagnosis code	Cases, n (%)
Addison's disease[2,3]	E27.1, E27.2, E27.4	320 (0.89)
Ankylosing spondylitis[4,5]	M45*	82 (0.23)
Autoimmune hepatitis[6]	K75.4	132 (0.37)
Crohn's disease[7,8]	K50*	502 (1.4)
Giant cell arteritis[9,10]	M31.5, M31.6	128 (0.36)
Glomerulonephritis[11]	N00*, N01*, N03*	229 (0.64)
Graves' disease[12,13]	E05.0*	589 (1.6)
Hashimoto's thyroiditis[12,13]	E06.3	1,165 (3.2)
Multiple sclerosis[14]	G35*	288 (0.80)
Myasthenia gravis[15]	G70*	93 (0.26)
Optic neuritis[16,17]	H46.0, H46.1, H46.8, H46.9	108 (0.30)
Polyarteritis nodosa[18]	M30*	18 (0.050)
Psoriasis[19,20]	L40*	916 (2.6)
Rheumatic mitral valve disease[21]	I05*	493 (1.4)
Sarcoidosis[22]	D86*	571 (1.6)
Type 1 diabetes[23]	E10*	968 (2.7)
Ulcerative colitis[7,8]	K51*	370 (1.0)
Vitiligo[24]	L80	175 (0.49)

ICD-10, International Classification of Diseases 10; n, number; *, indicates any ICD-10 code below the stated parent level (e.g., L40* includes L40.0, L40.1, L40.2, L40.3, etc.).

References

1. Marc Overhage, J., Ryan, P. B., Reich, C. G., Hartzema, A. G. & Stang, P. E. Validation of a common data model for active safety surveillance research. *J. Am. Med. Informatics Assoc.* **19**, 54–60 (2012).
2. Skov, J. *et al.* Heritability of Addison's disease and prevalence of associated autoimmunity in a cohort of 112,100 Swedish twins. *Endocrine* **58**, 521–527 (2017).
3. Olafsson, A. S. nae. & Sigurjonsdottir, H. A. gust. Increasing Prevalence Of Addison Disease: Results From A Nationwide Study. *Endocr. Pract.* **22**, 30–35 (2016).
4. Walsh, J., Hunter, T., Schroeder, K., Sandoval, D. & Bolce, R. Trends in diagnostic prevalence and treatment patterns of male and female ankylosing spondylitis patients in the United States, 2006-2016. *BMC Rheumatol.* **3**, (2019).
5. Lindström, U. *et al.* Validity of ankylosing spondylitis and undifferentiated spondyloarthritis diagnoses in the Swedish National Patient Register. *Scand. J. Rheumatol.* **44**, 369–376 (2015).
6. Kim, B. H. *et al.* Population-based prevalence, incidence, and disease burden of autoimmune hepatitis in South Korea. *PLoS One* **12**, (2017).
7. Stepaniuk, P., Bernstein, C. N., Nugent, Z. & Singh, H. Characterization of inflammatory bowel disease in hospitalized elderly patients in a large central Canadian health region. *Can. J. Gastroenterol. Hepatol.* **29**, 274 (2015).

8. Xu, F., Wheaton, A. G., Liu, Y., Lu, H. & Greenlund, K. J. Hospitalizations for Inflammatory Bowel Disease Among Medicare Fee-for-Service Beneficiaries — United States, 1999–2017. *Morb. Mortal. Wkly. Rep.* **68**, 1134–1138 (2019).
9. Aouba, A. *et al.* Mortality causes and trends associated with giant cell arteritis: Analysis of the French national death certificate database (1980-2011). *Rheumatol. (United Kingdom)* **57**, 1047–1055 (2018).
10. Wiberg, F., Naderi, N., Mohammad, A. J. & Turesson, C. Evaluation of revised classification criteria for giant cell arteritis and its clinical phenotypes. *Rheumatol. (United Kingdom)* **61**, 383–387 (2022).
11. Guo, Q., Wu, S., Xu, C. P., Wang, J. & Chen, J. Global Disease Burden From Acute Glomerulonephritis 1990–2019. *Kidney Int. Reports* **6**, 2212–2217 (2021).
12. Jølving, L. R. *et al.* Chronic diseases in the children of women with maternal thyroid dysfunction: A nationwide cohort study. *Clin. Epidemiol.* **10**, 1381–1390 (2018).
13. Song, Y. S., Kim, K. S., Kim, S. K., Cho, Y. W. & Choi, H. G. Screening leads to overestimated associations of thyroid dysfunction and thyroiditis with thyroid cancer risk. *Cancers (Basel).* **13**, 5385 (2021).
14. St. Germaine-Smith, C. *et al.* Recommendations for optimal ICD codes to study neurologic conditions a systematic review. *Neurology* **79**, 1049–1055 (2012).
15. Chen, J. *et al.* Incidence, mortality, and economic burden of myasthenia gravis in China: A nationwide population-based study. *Lancet Reg. Heal. - West. Pacific* **5**, 100063 (2020).
16. Nien, C. W. *et al.* The development of optic neuropathy after chronic rhinosinusitis: A population-based cohort study. *PLoS One* **14**, (2019).
17. Gu, W. *et al.* Incidence of Optic Neuritis and the Associated Risk of Multiple Sclerosis for Service Members of U.S. Armed Forces. *Mil. Med.* (2021) doi:10.1093/milmed/usab352.
18. Gokhale, M. *et al.* Prevalence of Eosinophilic Granulomatosis With Polyangiitis and Associated Health Care Utilization Among Patients With Concomitant Asthma in US Commercial Claims Database. *J. Clin. Rheumatol.* **27**, 107–113 (2021).
19. Koch, M. *et al.* Psoriasis and cardiometabolic traits: Modest association but distinct genetic architectures. *J. Invest. Dermatol.* **135**, 1283–1293 (2015).
20. Duvetorp, A., Mrowietz, U., Nilsson, M. & Seifert, O. Sex and Age Influence the Associated Risk of Depression in Patients with Psoriasis: A Retrospective Population Study Based on Diagnosis and Drug-Use. *Dermatology* **237**, 595–602 (2021).
21. Auckland, K. *et al.* The Human Leukocyte Antigen Locus and Rheumatic Heart Disease Susceptibility in South Asians and Europeans. *Sci. Rep.* **10**, 1–9 (2020).
22. Martusewicz-Boros, M. M., Boros, P. W., Wiatr, E., Fijołek, J. & Roszkowski-Śliż, K. Systemic treatment for sarcoidosis was needed for 16% of 1810 Caucasian patients. *Clin. Respir. J.* **12**, 1367–1371 (2018).
23. Dugan, J. & Shubrook, J. International Classification of Diseases, 10th Revision, Coding for Diabetes. *Clin. Diabetes* **35**, 232–238 (2017).
24. Lee, Y. B. & Kim, H. S. Height and risk of vitiligo: A nationwide cohort study. *J. Clin. Med.* **10**, (2021).
