# Supplementary material for: A machine learning model identifies patients in need of autoimmune disease testing using electronic health records
Source: Nat Commun. 2023 Apr 25;14:2385. doi: 10.1038/s41467-023-37996-7 (PMC10130143; doi:10.1038/s41467-023-37996-7)
Supplement: Supplementary file 2 — Reporting Summary [file 41467_2023_37996_MOESM2_ESM.pdf]

nature portfolio

Corresponding author(s): Ron Do

Last updated by author(s): 2023-3-27

Reporting Summary

Nature Portfolio wishes to improve the reproducibility of the work that we publish. This form provides structure for consistency and transparency in reporting. For further information on Nature Portfolio policies, see our Editorial Policies and the Editorial Policy Checklist.

Statistics

For all statistical analyses, confirm that the following items are present in the figure legend, table legend, main text, or Methods section.

n/a	Confirmed	
☐	☒	The exact sample size (*n*) for each experimental group/condition, given as a discrete number and unit of measurement
☐	☒	A statement on whether measurements were taken from distinct samples or whether the same sample was measured repeatedly
☐	☒	The statistical test(s) used AND whether they are one- or two-sided<br>*Only common tests should be described solely by name; describe more complex techniques in the Methods section.*
☐	☒	A description of all covariates tested
☐	☒	A description of any assumptions or corrections, such as tests of normality and adjustment for multiple comparisons
☐	☒	A full description of the statistical parameters including central tendency (e.g. means) or other basic estimates (e.g. regression coefficient) AND variation (e.g. standard deviation) or associated estimates of uncertainty (e.g. confidence intervals)
☐	☒	For null hypothesis testing, the test statistic (e.g. *F*, *t*, *r*) with confidence intervals, effect sizes, degrees of freedom and *P* value noted<br>*Give P values as exact values whenever suitable.*
☒	☐	For Bayesian analysis, information on the choice of priors and Markov chain Monte Carlo settings
☒	☐	For hierarchical and complex designs, identification of the appropriate level for tests and full reporting of outcomes
☐	☒	Estimates of effect sizes (e.g. Cohen's *d*, Pearson's *r*), indicating how they were calculated

Our web collection on statistics for biologists contains articles on many of the points above.

Software and code

Policy information about availability of computer code

Data collection	No software was used for the collection of data, as this was an opportunistic study.
Data analysis	Code for running and analyzing the machine learning model is available at https://data.mendeley.com/datasets/chg348gtxp/1.<br>All plots and statistical tests were generated with R (version 3.5.3).<br>Plots were produced using the pROC (version 1.16.2) and ggplot2 (version 3.3.3) packages, missing values were imputed via random forest-based algorithm using the missForest (version 1.4) package, features were selected with the Boruta function from the Boruta package (version 7.0.0), and the machine learning model was trained and tested using the caret (version 6.0.84) and randomForest (version 4.6-14) packages

For manuscripts utilizing custom algorithms or software that are central to the research but not yet described in published literature, software must be made available to editors and reviewers. We strongly encourage code deposition in a community repository (e.g. GitHub). See the Nature Portfolio guidelines for submitting code & software for further information.

Data

Policy information about availability of data

All manuscripts must include a data availability statement. This statement should provide the following information, where applicable:

- Accession codes, unique identifiers, or web links for publicly available datasets
- A description of any restrictions on data availability
- For clinical datasets or third party data, please ensure that the statement adheres to our policy

Data from All of Us is available via application to the Researcher Workbench at https://workbench.researchallofus.org/login. Further information regarding the BioMe Biobank and its dataset are available at https://icahn.mssm.edu/research/ipm/programs/biome-biobank, and further information regarding the Mount Sinai Data Warehouse and its dataset are available at https://labs.icahn.mssm.edu/msdw/data-sources. Access to these data needs to be requested from the BioMe Biobank and Mount Sinai Data Warehouse.

Human research participants

Policy information about studies involving human research participants and Sex and Gender in Research.

Reporting on sex and gender	In all analyses, biological sex was used. Distributions of sex are reported for each cohort in the Results section, and in autoantibody tested and not tested groups in Table 1.
Population characteristics	Given that this was an opportunistic study, participants were not selected based on any specific trait or disease. Population characteristics are described in the "Study population" subsection of the Results The population included 161,584 participants from three cohorts across two institutions (see Table 1 and Fig 1A). There were 25,062 participants in the BioMe Biobank (BioMe) cohort 1 (median [IQR] age, 60 [24] years; 15,091 [60%] female; 17,958 [72%] non-European ethnicity), comprising 6,171 (25%) individuals who had received autoantibody testing. There were 136,522 participants in All of Us (median [IQR] age, 61 [24] years; 85,196 [62%] female; 62,199 [46%] non-European ethnicity), including 19,264 (14%) individuals who had been tested for autoantibodies. There were 10,839 EHRs participants in BioMe cohort 2 (median [IQR] age, 56 [27] years; 6,243 [58%] female; 7,383 [68%] non-European ethnicity) used for clinical applications of the model.
Recruitment	This was an opportunistic secondary use study and therefore did not involve the recruitment of any participants.
Ethics oversight	The study protocols were approved by the Institutional Review Board at the Icahn School of Medicine at Mount Sinai and informed consent was obtained for all participants. Analyses of All of Us were completed according to the All of Us Code of Conduct and all participants provided informed consent; reported results comply with the All of Us Data and Statistics Dissemination Policy and are presented in groups of at least 20 individuals.

Note that full information on the approval of the study protocol must also be provided in the manuscript.

Field-specific reporting

Please select the one below that is the best fit for your research. If you are not sure, read the appropriate sections before making your selection.

[x] Life sciences   [ ] Behavioural & social sciences   [ ] Ecological, evolutionary & environmental sciences

For a reference copy of the document with all sections, see nature.com/documents/nr-reporting-summary-flat.pdf

Life sciences study design

All studies must disclose on these points even when the disclosure is negative.

Sample size	This study was opportunistic and involved secondary use of existing electronic health record data, and therefore no sample size was predetermined.<br>The current sample size enabled the accurate performance of the machine learning model with AUROC of 0.93 and 0.87 in the validation and external test sets, respectively (see Figure 1 and Table 2).
Data exclusions	Participant selection is described extensively in the Methods section and shown in detail in Supplementary Fig. 3.<br>Participants at least 20 years of age with at least 1 year of EHR data and 3 documented clinical encounters were selected (i.e., participants younger than 20 years of age, with less than 1 year of EHR data, or with less than 3 clinical encounters were excluded) to ensure cases and controls had sufficient EHR data for training and evaluating the model.
Replication	We externally tested the machine learning model in an external dataset from All of Us as described in the Methods section. The model had accurate performance in the external test dataset with AUROC of 0.87 (see Figure 1 and Table 2)
Randomization	This was a population-based study (not a case-control study) and thus no randomization was performed. All statistical analyses were adjusted for covariates of age, sex, BMI, and self-reported ethnicity as described in the "Statisical analysis" subsection of the Methods, unless stated otherwise in the text.

Blinding	As this was an observational population-based study (not a case-control study) blinding was not relevant Participants accrued clinical datapoints longitudinally in their health records agnostic to specific diseases or conditions. Large number of variables were collected and it not feasible to blind investigators to all aspects of the data being analyzed. Additionally, the observational nature of the study cohorts means that participants are not randomly assigned to different treatment groups.

Reporting for specific materials, systems and methods

We require information from authors about some types of materials, experimental systems and methods used in many studies. Here, indicate whether each material, system or method listed is relevant to your study. If you are not sure if a list item applies to your research, read the appropriate section before selecting a response.

Materials & experimental systems

n/a	Involved in the study
☒	☐ Antibodies
☒	☐ Eukaryotic cell lines
☒	☐ Palaeontology and archaeology
☒	☐ Animals and other organisms
☒	☐ Clinical data
☒	☐ Dual use research of concern

Methods

n/a	Involved in the study
☒	☐ ChIP-seq
☒	☐ Flow cytometry
☒	☐ MRI-based neuroimaging
